# Incoherent excess noise spectrally encodes broadband light sources

**DOI:** 10.1038/s41377-020-00404-6

**Published:** 2020-10-06

**Authors:** Aaron M. Kho, Tingwei Zhang, Jun Zhu, Conrad W. Merkle, Vivek J. Srinivasan

**Affiliations:** 1 Department of Biomedical Engineering, University of California Davis, Davis, CA 95616 USA; 2 Department of Ophthalmology and Vision Science, University of California Davis School of Medicine, Sacramento, CA 95817 USA

**Keywords:** Optical spectroscopy, Biophotonics, Imaging and sensing

## Abstract

Across optics and photonics, excess intensity noise is often considered a liability. Here, we show that excess noise in broadband supercontinuum and superluminescent diode light sources encodes each spectral channel with unique intensity fluctuations, which actually serve a useful purpose. Specifically, we report that excess noise correlations can both characterize the spectral resolution of spectrometers and enable cross-calibration of their wavelengths across a broad bandwidth. Relative to previous methods that use broadband interferometry and narrow linewidth lasers to characterize and calibrate spectrometers, our approach is simple, comprehensive, and rapid enough to be deployed during spectrometer alignment. First, we employ this approach to aid alignment and reduce the depth-dependent degradation of the sensitivity and axial resolution in a spectrometer-based optical coherence tomography (OCT) system, revealing a new outer retinal band. Second, we achieve a pixel-to-pixel correspondence between two otherwise disparate spectrometers, enabling a robust comparison of their respective measurements. Thus, excess intensity noise has useful applications in optics and photonics.

Optical spectrometers, which measure light intensity on a wavelength-by-wavelength basis, benefit many fields, including biomedical science^1–3^, agriculture^4^, and security^5^. Spectral resolution refers to the ability of a spectrometer to distinguish fine spectral features. In a common spectrometer design that disperses light across a detector array (Fig. 1a), the spectral resolution is ideally determined by the equivalent spectral widths of the sensor pixels and dispersive element resolution^6,7^. Pixel cross-talk and optical aberrations, including those caused by misalignment of refractive elements, reflective elements, diffractive elements, and the sensor, all degrade the spectral resolution (see Supplementary Note 1 for a more complete discussion of spectrometer performance). While simulations can determine idealized positioning of optical components, in practice, optimal placement of components a priori is difficult given manufacturing tolerances, and alignment is still required (see Supplementary Note 2). Feedback on the spectral resolution across the entire spectrometer during alignment is highly desirable but currently impractical, as described below. Moreover, for homebuilt and commercial spectrometers with varying specifications, cross-calibration of wavelengths is needed to compare spectral features, such as Raman peaks, which relate to the chemical composition^3^. The alignment and specifications may change as spectrometers experience wear-and-tear. Overall, to improve the rigor and reproducibility of research that uses spectrometers, we identify two unmet needs: first, rapid and comprehensive characterization of the spectrometer resolution, and second, pixel-by-pixel calibration of spectrometer wavelengths. Current approaches for spectrometer characterization and calibration^8^ are slow, cumbersome, or limited in spectral range.

**Fig. 1:**
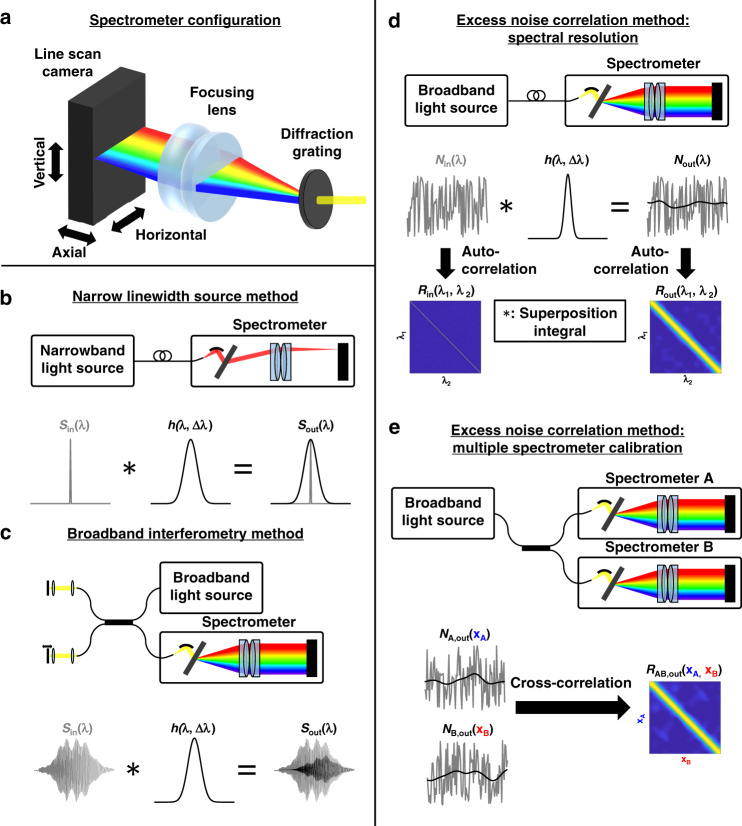
Spectrometer characterization and calibration methods. **a** Typical spectrometer. Collimated light is spectrally dispersed by the diffraction grating and focused onto a linear sensor. **b** The narrow linewidth source method requires a narrowband light source for each wavelength to be assessed (top). The measured spectrum output, *S*_*out*_(*λ*), is the superposition integral of the true spectrum input, *S*_*in*_(*λ*), and the spectrometer impulse response, *h*(*λ*, Δ*λ*): $S_{out}\left( \lambda \right) = {\int} {S_{in}} \left( {\mathrm{{\Lambda} }} \right)h\left( {{\mathrm{{\Lambda} }},\lambda - {\mathrm{{\Lambda} }}} \right)d{\mathrm{{\Lambda} }}$. If the input approximates a delta function, then the output, *S*_*out*_(*λ*), resembles *h*(*λ*, Δ*λ*) (bottom). **c** The broadband interferometry method requires an auxiliary interferometer to create an oscillating interferometric input, *S*_*in*_(*λ*) (top). The spectrometer reduces the oscillations in the output, *S*_*out*_(*λ*), depending on the impulse response (bottom). See Supplementary Note 6 for a complete description. **d** In the proposed excess noise method for characterization, an appropriate broadband light source is required (top). The output, *N*_*out*_(*λ*), is the superposition integral of the excess noise input, *N*_*in*_(*λ*), and *h*(*λ*, Δ*λ*). For white noise input, the input autocorrelation matrix, *R*_*in*_(*λ*_1_, *λ*_2_), is diagonal. The output autocorrelation matrix, *R*_*out*_(*λ*_1_, *λ*_2_), is quasi-diagonal, with broadening depending on the local impulse response (bottom). **e** In the related method for cross-calibration, an appropriate broadband light source and a coupler are required (top). The excess noise outputs from spectrometers A and B, *N*_*A,out*_(*x*_*A*_) and *N*_*B,out*_(*x*_*B*_), respectively, are cross-correlated to yield *R*_*AB,out*_(*x*_*A*_, *x*_*B*_), where the highest correlation values occur for pixels that measure similar wavelengths (bottom)

To provide an approach to address these problems, we turn to a somewhat unexpected phenomenon: the excess noise in broadband light sources^9–12^. “Excess” refers to noise in excess of fundamental quantum shot noise. Interestingly, in photonics applications described heretofore, excess noise degraded the performance. We show that, rather than being a liability, excess noise imbues broadband light with high-resolution spectral encoding (see Supplementary Note 3), which is a natural conduit for spectrometer characterization and cross-calibration. Based on this insight, we develop a simple strategy to characterize the spectral resolution of spectrometers. We also develop an approach to create a precise mapping between pixels of two different spectrometers that correspond in wavelength, hereafter referred to as cross-calibration. We validate our approach against conventional methods across multiple spectral ranges, showing its broad applicability to both supercontinuum and superluminescent diode light sources. We then demonstrate its utility by improving the spectral resolution of multiple visible light optical coherence tomography (OCT) spectrometers and visualizing a new band in the mouse outer retina. Next, we demonstrate cross-calibration of two otherwise disparate spectrometers with high accuracy. Thus, our method for spectrometer characterization and cross-calibration represents a unique application of excess noise.

Assessing the spectral resolution, or spectrometer characterization, essentially reduces to system identification^7,8^. Two methods are currently used^8^. The first, the impulse response method, determines the spectral resolution from the measured intensity pattern of a narrow linewidth light source or sharp spectral feature, ideally with a lineshape much narrower than the spectral resolution (Fig. 1b). However, this approach requires additional narrow linewidth sources, narrowband optical filters, or sources with fine spectral features (e.g., Fraunhofer lines or frequency combs). For a comprehensive characterization, fine spectral features are required at each measured wavelength. While a tuneable, narrow linewidth source could provide a universal approach, such sources are not available for all wavelength ranges (e.g., visible). The second, the transfer function method, determines the spectral resolution across wavelengths from the attenuation of a sinusoidal interference fringe pattern envelope as the path length mismatch increases (Fig. 1c). This method can yield the spectral resolution for every sensor pixel, but only if the spectral resolution is slowly varying across the sensor. Data acquisition can be time consuming, requiring multiple measurements with an external, variable path length interferometer. Thus, neither of the two existing characterization methods are practical during spectrometer alignment.

Similarly, for the related problem of assigning pixels to wavelengths, or spectrometer calibration, current approaches utilize either fine optical features, such as narrowband lasers or spectral lines with well-known and invariant wavelengths, or interferometry^13,14^. If the spectrometer is previously calibrated at one or more pixels, then broadband interferometry with highly accurate path length variations can be used to calibrate the remainder^15,16^. Otherwise, a tuneable narrow linewidth source is required at all wavelengths. Thus, a simple method for pixel-by-pixel spectrometer characterization and calibration remains elusive.

Here, we present a method that provides both the spectral resolution and wavelength correspondence between spectrometers from a single time series of noise registered by different sensor pixels. If the intrinsic source noise is incoherent or uncorrelated between wavelengths separated on the scale of the spectral resolution, then any measured excess noise correlation between pixels is attributable to the nonzero spectral resolution (Fig. 1d and Supplementary Note 3), which causes those pixels to measure similar wavelengths (see Supplementary Note 4 for a discussion of the applicability when this assumption is violated). The local extent of this measured correlation across pixels in the spectrometer relates to the spectral resolution. The conditions responsible for spectrally uncorrelated or “incoherent” noise are briefly described in Supplementary Note 5.

The excess noise autocorrelation matrix (Fig. 2a), estimated from individual pixel time courses and corrected for shot and detector noise (comprising dark noise and read noise), is the basis of our method for characterizing the spectral resolution (see Supplementary Note 3). Shot noise must be uncorrelated between pixels, with a variance proportional to the pixel gray level, while the excess noise variance goes as the square of the gray level. While relative intensity noise (RIN) and excess noise are sometimes used interchangeably^17^, this usage is not universal^7,18,19^, and we will refrain from discussing RIN. Practically, we can distinguish shot noise and detector noise from excess noise based on the quadratic light intensity dependence of the latter. Pairs of pixels with high excess noise correlations are observed along a quasi-diagonal region (Fig. 2a subplots). A broader quasi-diagonal width, seen here at the edges of the sensor (lower right of the matrix), implies degraded spectral resolution (Fig. 2b) compared to the narrower quasi-diagonal width towards the middle of the sensor (center of the matrix). For a visible light OCT spectrometer used for mouse retinal imaging^20^, spectral resolutions from the excess noise method were directly compared to those obtained from both narrow linewidth (Fig. 1b) and interferometry (Fig. 1c) methods (see Supplementary Note 6), which required additional narrowband light sources and an auxiliary interferometer, respectively. The three methods agree well across most of the spectrometer range (Fig. 2b), supporting the validity of the excess noise method. The methods disagree at the edges of the spectral range due to the low intensities and insufficient excess noise. The excess noise of an infrared (NIR-II) superluminescent diode, though more than two orders of magnitude smaller than that of the visible supercontinuum (see Supplementary Note 7), was further employed to characterize two spectrometers, yielding results in agreement with interferometry (see Supplementary Note 8).

**Fig. 2:**
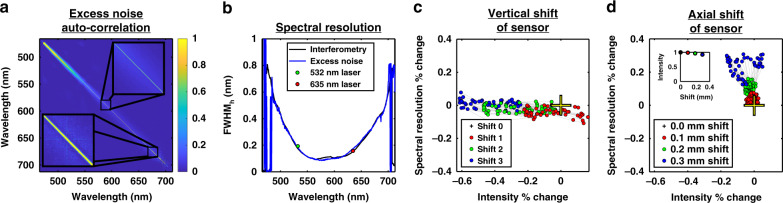
Excess noise autocorrelation can characterize spectrometers. **a** Excess noise autocorrelation matrix from a previously reported visible light OCT spectrometer^20^. The zoom-ins of the autocorrelation matrix show a thinner quasi-diagonal at central wavelengths than at peripheral wavelengths in the spectrum. **b** The spectral resolution measured from this autocorrelation matrix with the proposed method agrees well with the conventional interferometry and narrowband laser calibration method results. **c** Vertical shifting of the sensor (as depicted in Fig. 1a), relative to the optimal position, mainly changes the intensity measured by the pixels (dots). Shift 1 denotes the smallest shift, while shift 3 denotes the largest shift from the optimal position. Due to the small magnitude of the shift relative to the translation stage screw pitch, the shifts were not precisely measured. **d** Axial shifting of the sensor (as depicted in Fig. 1a) towards the focusing lens, relative to the optimal position, mainly changes the spectral resolution. The subplot shows the summed total spectrum intensity for each shift normalized to the total spectrum intensity at the optimal position

Returning to the visible light OCT spectrometer, the sensor was then deliberately misaligned by translation in the vertical and axial directions (Fig. 1a) to investigate the effects on both the spectral resolution and intensity (taken as the pixel gray level). Vertical misalignment results in the focused line missing the sensor, reducing the intensity (Fig. 2c), while axial misalignment defocuses the light hitting the sensor, reducing the intensity and degrading the spectral resolution. Due to our asymmetric pixel size of 10 × 20 (horizontal × vertical) microns, the spectral resolution is far more sensitive than the intensity to axial misalignment (Fig. 2d). Thus, the intensity and spectral resolution are complimentary, and both are needed for accurate spectrometer alignment.

Next, we investigated further rearrangement of the optical components while monitoring the spectral resolution and intensity simultaneously. We improved our original spectrometer primarily by translating the focusing lens and sensor closer to the diffraction grating compared to the original back focal plane configuration (Fig. 3a, b). Although these two positions yielded very different spectral resolutions (Fig. 3c), they were essentially indistinguishable based on the conventional metrics of the spectral shape and intensity (Fig. 3c subplot). This is expected because aberrations, including defocusing, along the vertical plane (Fig. 1a) affect the registered intensity, while those along the horizontal plane (Fig. 1a) affect the spectral resolution. In this case, the asymmetric pixel size enabled improvement of the horizontal focus, i.e., spectral resolution, at the expense of the vertical focus, without compromising the intensity. The improved configuration homogenized the spectral resolution across the spectral range (Fig. 3c). The theoretical spectral resolution limit (see Supplementary Note 1) is shown for reference.

**Fig. 3:**
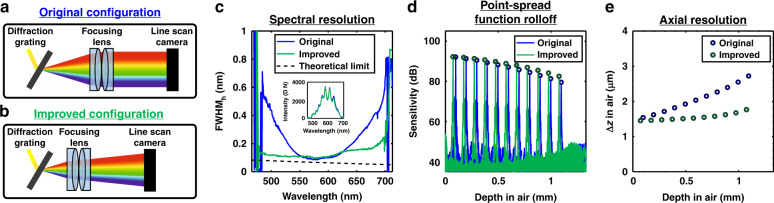
Quasi-real-time characterization improves the OCT spectrometer alignment. **a** Original spectrometer configuration with the diffraction grating at the back focal plane of the focusing lens. **b** Improved spectrometer configuration enabled by monitoring the spectral resolution during the alignment process. **c** The spectral resolution was noticeably more uniform across the spectrum in the improved configuration, though the measured spectrum intensities of both configurations, with an input power into the spectrometer of 1.55 *μ*W, were indistinguishable (inset). The OCT point spread function rolloff (**d**) and axial resolution degradation versus depth (**e**) demonstrate a marked improvement

For this spectrometer, designated henceforth as spectrometer A, the point spread function (PSF) rolloff improved to 8.7 dB, from 11.7 dB (Fig. 3d), and the axial resolution degradation improved to 18%, from 75% (Fig. 3e), over the first 1 mm of imaging depth in air (75% of the imaging range). The maximal sensitivity was virtually unchanged in the improved configuration. A change was noted in the noise floor rolloff, explainable through the Wiener–Khinchin theorem (see Supplementary Note 9). For another spectrometer, designated henceforth as spectrometer B, for human retinal imaging, a similar alignment procedure improved the PSF rolloff to 3.4 dB, from 6.3 dB, and improved the axial resolution degradation to 5%, from 43%, over the first 1 mm of imaging depth in air (48% of the imaging range) (see Supplementary Note 10). The near-uniform axial resolution arises from mitigation of the wavelength-dependent spectral resolution (wavenumber space), which mitigates the spectrally dependent rolloff. The improvement mechanism is confirmed by ray tracing simulations (see Supplementary Note 2).

When employed in a spectral/Fourier domain visible light OCT system (Fig. 4a), the improved spectrometer A helped visualize a hyporeflective band inner to the external limiting membrane (ELM) in the mouse retina (Fig. 4b, c). This band was found to possess different reflectivity than both the inner segments (IS) and outer nuclear layer (ONL) (Fig. 4d). Though situated in a stratum conventionally assigned to the ONL, a layer mostly composed of cell bodies, this hyporeflective band could represent a cell nuclei free layer inner to the junctional complexes that comprise the ELM observed in fluorescence microscopy^21^ and electron microscopy^22^. The reflectivity and regularity of this band could relate to photoreceptor or Müller cell health and organization. While several bands are more consistently resolved with visible light OCT than with near-infrared OCT, this thin hyporeflective band (Fig. 4e) is, to the best of our knowledge, the first new retinal feature revealed by visible light OCT.

**Fig. 4:**
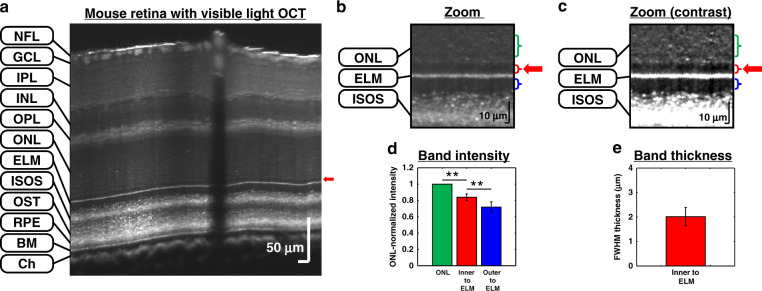
Visible light OCT visualizes a new outer retinal band with an improved spectrometer. **a** Cross-sectional linear-scaled image of a pigmented mouse retina, acquired by a visible light OCT system with a spectrometer aligned using excess noise correlations (Figs. 2 and 3). A total of 1024 frames, acquired over 17.5 s with a 0.12 mm offset along the slow axis, were averaged. The red arrow indicates a dark band inner to the ELM. **b** Linear-scaled, outer retinal zoomed-in view showing the newly visualized dark band (red arrow). **c** Contrast-enhanced zoomed-in view on a linear scale. **d** The ONL-normalized intensity of the dark band inner to the ELM (red brackets in **b**, **c**) is significantly different from 1 and from that of the inner segments (blue brackets in **b**, **c**) in six mice (The ONL region for normalization is denoted by green brackets in **b**, **c**). **e** The thickness of this dark band, taken as the FWHM of a fitted Gaussian, was ~2 μm in six mice. Error bars represent standard deviations across subjects (**p < 0.05). Note that no error bars are shown for the ONL in d due to normalization. (NFL: nerve fiber layer; GCL: ganglion cell layer; IPL: inner plexiform layer; INL: inner nuclear layer; OPL: outer plexiform layer; ONL: outer nuclear layer; ELM: external limiting membrane; ISOS: inner segment/outer segment junction; OST: photoreceptor outer segment tips; RPE: retinal pigment epithelium; BM: Bruch’s membrane; Ch: choroid)

The spectral encoding provided by excess noise also aids the cross-calibration of multiple spectrometers (Fig. 1e) via a cross-correlation matrix (Fig. 5a). While the rows and columns of the autocorrelation matrix represent pixels of the same spectrometer, the rows and columns of the cross-correlation matrix represent pixels of different spectrometers, with correlations calculated from synchronous time courses (see Supplementary Note 11). A high correlation indicates measurement of similar wavelengths. Therefore, for each pixel in one spectrometer, the pixel in the other with the maximum correlation is closest in wavelength (Fig. 5b).

**Fig. 5:**
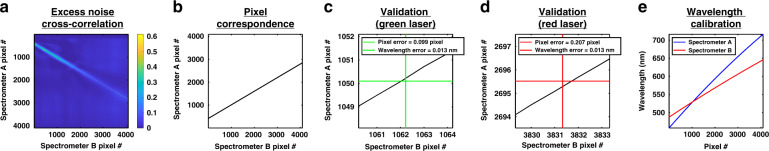
Excess noise cross-correlation can cross-calibrate spectrometers. **a** Excess noise cross-correlation matrix for two spectrometers with different spectral bandwidths: an already-calibrated spectrometer A (Figs. 2 and 3) and spectrometer B (see Supplementary Note 10), which had to be calibrated de nuovo. **b** Pixel correspondence between the two spectrometers. For each spectrometer A pixel (row), the spectrometer B pixel that yields the largest normalized excess noise cross-correlation matrix value (**a**) corresponds best in wavelength. The spectrometer A pixel position of maximum correlation for each spectrometer B pixel was estimated using Gaussian fitting for subpixel accuracy. **c**, **d** The inter-spectrometer calibration was validated with a green (~532 nm) laser and a red (~635 nm) laser, respectively, with subpixel estimates of the centroids of the laser distributions. The errors calculated as the shortest distances in pixels to the validation values (cross centers in **c**, **d**) range from 10–21% of a pixel. The errors calculated by the difference in assigned wavelengths are 0.013 nm for both narrowband lasers. **e** Thus, using this method, given wavelength calibration of spectrometer A, spectrometer B can be accurately calibrated

Inter-spectrometer calibration was validated by measuring the intensity distributions of narrowband green (~532 nm) and red (~635 nm) lasers on both spectrometers simultaneously. For each laser, the centroid was determined on both spectrometers, providing a subpixel correspondence that did not require knowledge of the exact wavelength. This two-point correspondence between spectrometers determined based on the narrowband lasers was compared to the comprehensive pixel-to-pixel correspondence from the excess noise correlation method (Fig. 5c, d). The pixel error, defined as the distance from the two designated corresponding points to the pixel calibration curve (Fig. 5c, d), was much less than 1. The difference in the assigned wavelengths for the two spectrometers (Fig. 5e) was 0.013 nm for both designated corresponding points.

The cross-calibration method does not, by itself, provide the absolute wavelengths of either spectrometer. However, this method can calibrate any spectrometer via another that was calibrated previously. For example, if the first spectrometer is calibrated and kept under controlled laboratory conditions while the second spectrometer is used in the field, the cross-calibration approach could be used to recalibrate the second spectrometer upon its return. We show that our method can calibrate a spectrometer *de nuovo* using another previously calibrated one via subpixel fitting and interpolation (Fig. 5e). Moreover, even in the absence of an absolute calibration, cross-calibration may improve the reproducibility of measurements taken by different spectrometers. The cross-calibration method could eventually improve the performance of OCT systems that employ multiple spectrometers^14,23^.

This work, to our knowledge, presents a novel and useful application of excess intensity noise in optics. Unlike previous approaches for characterizing and calibrating spectrometers, our excess noise correlation method is computationally simple, comprehensive, and fast (see Supplementary Note 12). It can be applied in situ, providing essential information to guide spectrometer alignment and determine the wavelength correspondence. This application benefits from higher levels of excess noise, which is characteristic of lower cost (lower repetition rate) supercontinuum sources. Notably, the idea fails with low noise sources based on pure self-phase modulation in nonlinear fibers and tungsten halogen lamps (see Supplementary Note 7) but applies to amplified spontaneous emission sources (see Supplementary Note 8). Though the finest measurable spectral resolution is ultimately limited by the intrinsic spectral correlations of the light source^11^, such a limitation was not detectable with either superluminescent or supercontinuum sources at spectral resolutions of 0.05–0.15 nm (see Supplementary Note 4). Our formalism can be modified to incorporate the intrinsic spectral correlations of the light source, if known, to mitigate this limitation (see Supplementary Note 4). Moreover, a modified fitting approach can exclude excess noise with a distinctly longer spectral correlation length to better isolate filtered incoherent noise to retrieve the spectral resolution (see Supplementary Note 3).

After the invention of the laser, the observed speckle pattern was initially viewed as a hindrance. However, temporal speckle correlations were later found to solve many problems in optics, providing information about blood flow and particle size, while spatial speckle correlations provided information about the diffraction limit of imaging systems^24,25^. Analogously, we hope that this work galvanizes the investigation of excess intensity noise correlations to solve other problems in optics.

## Methods

### Spectral resolution characterization with excess noise correlations

We assume that the spectrometer is a linear, but not necessarily wavelength shift-invariant, system such that $N_{out}\left( \lambda \right) = {\int} N_{in}\left( {\mathrm{{\Lambda} }} \right)h\left( {{\mathrm{{\Lambda} }},\lambda - {\mathrm{{\Lambda} }}} \right)d{\mathrm{{\Lambda} }}$, where *N*_*out*_(*λ*) and *N*_*in*_(*λ*) are the measured and intrinsic source excess noise, respectively, as a function of wavelength (*λ*). In this shift-variant linear system, *N*_*out*_(*λ*) is the superposition integral of *N*_*in*_(*λ*) and *h*(*λ*, Δ*λ*). The spectrometer impulse response function, *h*(*λ*, Δ*λ*), is a function of *λ*, the input wavelength, and Δ*λ*, the difference between the measured and input wavelengths. The first argument allows the impulse response to vary with wavelength. As *h*(*λ*, Δ*λ*) is a possible spectral intensity distribution (Fig. 1b), it must be nonnegative. Our proposed method utilizes the correlation of the excess noise, *R*_*out*_(*λ*_1_, *λ*_2_), between pairs of measured wavelengths, *λ*_1_ and *λ*_2_, to infer *h*(*λ*, Δ*λ*) and extract its width in Δ*λ*, known as the spectral resolution. The input (true) and output (measured) excess noise correlations are

1 $$R_{in}\left( {\lambda _1,\lambda _2} \right) = \left\langle {N_{in}\left( {\lambda _1} \right)N_{in}\left( {\lambda _2} \right)} \right\rangle \,{\mathrm{and}}$$

2 $$R_{out}\left( {\lambda _1,\lambda _2} \right) = \left\langle {N_{out}\left( {\lambda _1} \right)N_{out}\left( {\lambda _2} \right)} \right\rangle$$

respectively, where *N*_*in*_ is the zero-mean input excess noise and *N*_*out*_ is the zero-mean output excess noise. We take 〈·〉 to denote the ensemble average, which is estimated here by time averaging. All quantities are assumed to be real. Using the linear system assumption described above, Eq. (2) takes the form

3 $$\begin{array}{ll}R_{out}\left( {\lambda _1,\lambda _2} \right) = & \Bigl\langle \displaystyle{{\int} {N_{in}} \left( {{\mathrm{{\Lambda} }}_1} \right)h\left( {{\mathrm{{\Lambda} }}_1,\lambda _1 - {\mathrm{{\Lambda} }}_1} \right)d{\mathrm{{\Lambda} }}_1\displaystyle{\int} {N_{in}} \left( {{\mathrm{{\Lambda} }}_2} \right)}\Bigr.\\& \Bigl.{ h\left( {{\mathrm{{\Lambda} }}_2,\lambda _2 - {\mathrm{{\Lambda} }}_2} \right)d{\mathrm{{\Lambda} }}_2}\Bigr\rangle\end{array}$$

We can then substitute Eq. (1) into Eq. (3), using the fact that *h* is invariant, to obtain *R*_*out*_(*λ*_1_, *λ*_2_) in terms of *R*_*in*_(*λ*_1_, *λ*_2_):

4 $$\begin{array}{ll}R_{out}\left( {\lambda _1,\lambda _2} \right) =& \displaystyle{\int\!\!\!\!\!\int} {R_{in}} \left( {{\mathrm{{\Lambda} }}_1,{\mathrm{{\Lambda} }}_2} \right)h\left( {{\mathrm{{\Lambda} }}_1,\lambda _1 - {\mathrm{{\Lambda} }}_1} \right)d{\mathrm{{\Lambda} }}_1\\&h\left( {{\mathrm{{\Lambda} }}_2,\lambda _2 - {\mathrm{{\Lambda} }}_2} \right)d{\mathrm{{\Lambda} }}_2\end{array}$$

Assuming that the excess noise is white, we can express *R*_*in*_(*λ*_1_, *λ*_2_) as a delta function, *δ*(*λ*_2_ − *λ*_1_). Thus,

5 $$R_{in}\left( {\lambda _1,\lambda _2} \right) = \sigma ^2\left( {\lambda _1} \right)\delta \left( {\lambda _2 - \lambda _1} \right)$$

where *σ*^2^(*λ*) is the wavelength-dependent variance. In cases where the white noise assumption is invalid^11^, the above expression can be modified to accommodate a nonimpulsive *R*_*in*_(see Supplementary Note 4). Substituting Eq. (5) into Eq. (4) yields

6 $$\begin{array}{ll}R_{out}\left( {\lambda _1,\lambda _2} \right) = &\displaystyle{\int} {\sigma ^2} \left( {{\mathrm{{\Lambda} }}_1} \right)h\left( {{\mathrm{{\Lambda} }}_1,\lambda _1 - {\mathrm{{\Lambda} }}_1} \right)\\& h\left( {{\mathrm{{\Lambda} }}_1,\lambda _2 - {\mathrm{{\Lambda} }}_1} \right)d{\mathrm{{\Lambda} }}_1\end{array}$$

Assuming that *σ*^2^(*λ*) varies slowly on the scale of the spectral resolution, we can remove it from the integral:

7 $$\begin{array}{ll}R_{out}\left( {\lambda _1,\lambda _2} \right) = & \sigma ^2\left( {\frac{{\lambda _1 + \lambda _2}}{2}} \right)\displaystyle{\int} h \left( {{\mathrm{{\Lambda} }}_1,\lambda _1 - {\mathrm{{\Lambda} }}_1} \right)\\& h\left( {{\mathrm{{\Lambda} }}_1,\lambda _2 - {\mathrm{{\Lambda} }}_1} \right)d{\mathrm{{\Lambda} }}_1\end{array}$$

We can further simplify Eq. (7) by using the substitutions ${\mathrm{{\Lambda} }}^\prime = \lambda _1 - {\mathrm{{\Lambda} }}_1$ and ${\mathrm{{\Delta} }}\lambda = \lambda _2 - \lambda _1$:

8 $$\begin{array}{ll}R_{out}\left( {\lambda _1,\lambda _2} \right) = & \sigma ^2\left( {\frac{{\lambda _1 + \lambda _2}}{2}} \right)\displaystyle{\int} h \left( {{\uplambda}_1 - {\mathrm{{\Lambda} }}^\prime ,{\mathrm{{\Lambda} }}^\prime } \right)\\ {}& h\left( {{\uplambda}_1 - {\mathrm{{\Lambda} }}^\prime ,{\mathrm{{\Lambda} }}^\prime + {\mathrm{{\Delta} }}\lambda } \right)d{\mathrm{{\Lambda} }}^\prime\end{array}$$

If *h*(*λ*, Δ*λ*) varies slowly in *λ* compared to Δ*λ*, then Eq. (8) can take the form

9 $$R_{out}\left( {\lambda _1,\lambda _2} \right) = \sigma ^2\left( {\lambda _{avg}} \right)h\left( {\lambda _{avg},{\mathrm{{\Delta} }}\lambda } \right) \star h\left( {\lambda _{avg},{\mathrm{{\Delta} }}\lambda } \right)$$

where $\lambda _{avg} = \frac{{\lambda _1 + \lambda _2}}{2},{\mathrm{{\Delta} }}\lambda = \lambda _2 - \lambda _1$, and $\star$ denotes the cross-correlation with respect to Δ*λ*. This leads to the natural reparameterization $R_{out}^\prime \left( {\lambda _{avg},{\mathrm{{\Delta} }}\lambda } \right) = R_{out}\left( {\lambda _{avg} - \frac{{{\mathrm{{\Delta} }}\lambda }}{2},\lambda _{avg} + \frac{{{\mathrm{{\Delta} }}\lambda }}{2}} \right)$. If we assume a Gaussian impulse response (see Supplementary Note 1), i.e.,

10 $$h\left( {\lambda _{avg},{\mathrm{{\Delta} }}\lambda } \right)\sim {\cal{N}}\left[ {0,\sigma _\lambda ^2\left( {\lambda _{avg}} \right)} \right]$$

where ${\cal{N}}$ denotes a normal distribution with zero mean and a variance of $\sigma _\lambda ^2\left( {\lambda _{avg}} \right)$, then

11 $$h\left( {\lambda _{avg},{\mathrm{{\Delta} }}\lambda } \right) \star h\left( {\lambda _{avg},{\mathrm{{\Delta} }}\lambda } \right)\sim {\cal{N}}\left[ {0,2\sigma _\lambda ^2\left( {\lambda _{avg}} \right)} \right]$$

By using Eqs. (10) and (11), we find the relationship between the full-width-at-half-maximum (FWHM) of *h*(*λ*_*avg*_, Δ*λ*) and the FWHM of $R_{out}^\prime \left( {\lambda _{avg},{\mathrm{{\Delta} }}\lambda } \right)$ to be

12 $$FWHM_h\left( {\lambda _{avg}} \right) = \frac{{FWHM_{R_{out}^\prime }\left( {\lambda _{avg}} \right)}}{{\sqrt 2 }}$$

Therefore, we can find the desired FWHM spectral resolution, *FWHM*_*h*_(*λ*_*avg*_), by analysing the excess noise autocorrelation matrix, *R*_*out*_(*λ*_1_, *λ*_2_), either directly or in normalized form (Supplementary Note 3).

### Spectrometer cross-calibration with excess noise correlations

To describe the cross-calibration of spectrometers A and B, we express the input and output excess noise correlations as

13 $$R_{in}\left( {\lambda _1,\lambda _2} \right) = \left\langle {N_{in}\left( {\lambda _1} \right)N_{in}\left( {\lambda _2} \right)} \right\rangle \,{\mathrm{and}}$$

14 $$R_{AB,out}\left( {x_A,x_B} \right) = \left\langle {N_{A,out}\left( {x_A} \right)N_{B,out}\left( {x_B} \right)} \right\rangle$$

respectively, where *N*_*in*_(*λ*), *N*_*A,out*_(*x*_*A*_) and *N*_*B,out*_(*x*_*B*_) are the zero-mean real-valued input and output excess noise of spectrometers A and B. Note that the premise of the cross-calibration method is that the spectrometer wavelengths are unknown *a priori*, so we assume that *R*_*AB,out*_(*x*_*A*_, *x*_*B*_) is a function of the chosen pixels, *x*_*A*_ and *x*_*B*_, in the respective spectrometers. Spectrometers A and B are assumed to have partially overlapping wavelength ranges but unknown pixel-to-wavelength mappings, *λ*_*A*_(*x*_*A*_) and *λ*_*B*_(*x*_*B*_), which can be determined through a separate procedure.

As above, from the linear shift-variant system assumption, Eq. (14) becomes

15 $$R_{AB,out}\left( {x_A,x_B} \right) = \left\langle \int {N_{in}} \left({\mathrm{{\Lambda} }}_A \right)h_A\left[{{\mathrm{{\Lambda} }}_A,\lambda _A\left( {x_A} \right) -{\mathrm{{\Lambda} }}_A} \right]d{\mathrm{{\Lambda} }}_A{\int}{N_{in}} \left( {{\mathrm{{\Lambda} }}_B} \right)h_B\left[{{\mathrm{{\Lambda} }}_B,\lambda _B\left( {x_B} \right) -{\mathrm{{\Lambda} }}_B} \right]d{\mathrm{{\Lambda} }}_B \right\rangle$$

Equation (15) can be expressed in terms of *R*_*in*_(*λ*_*A*_, *λ*_*B*_) using Eq. (13):

16 $$R_{AB,out}\left( {x_A,x_B} \right) = {\int\!\!\!\!\!\int} {R_{in}} \left( {{\mathrm{{\Lambda} }}_A,{\mathrm{{\Lambda} }}_B} \right)h_A\left[ {{\mathrm{{\Lambda} }}_A,\lambda _A\left( {x_A} \right) - {\mathrm{{\Lambda} }}_A} \right]d{\mathrm{{\Lambda} }}_A h_B\left[ {{\mathrm{{\Lambda} }}_B,\lambda _B\left( {x_B} \right) - {\mathrm{{\Lambda} }}_B} \right]d{\mathrm{{\Lambda} }}_B$$

Assuming excess noise is white, we can express the input excess noise correlation as Eq. (5). Using Eq. (5) and assuming that *σ*^2^(*λ*) varies slowly compared to the spectral resolution, Eq. (16) becomes

17 $$R_{AB,out}\left( {x_A,x_B} \right) = \sigma ^2\left\{ {\frac{{\lambda _A\left( {x_A} \right) + \lambda _B\left( {x_B} \right)}}{2}} \right\}{\int} {h_A} \left[ {{\mathrm{{\Lambda} }}_A,\lambda _A\left( {x_A} \right) - {\mathrm{{\Lambda} }}_A} \right]h_B\left[ {{\mathrm{{\Lambda} }}_A,\lambda _B\left( {x_B} \right) - {\mathrm{{\Lambda} }}_A} \right]d{\mathrm{{\Lambda} }}_A$$

For a given pixel on spectrometer A, *x*_*A*_, the cross-correlation *R*_*AB,out*_(*x*_*A*_, *x*_*B*_) achieves a maximum when both impulse response functions, *h*_*A*_ and *h*_*B*_, share the same maximum with respect to Λ_*A*_ in Eq. (17). Therefore, the output excess noise correlation, *R*_*AB,out*_(*x*_*A*_, *x*_*B*_), is maximized when *λ*_*A*_(*x*_*A*_) = *λ*_*B*_(*x*_*B*_), i.e., when the pixels measure the same wavelength. Even if the excess noise is not white, this conclusion remains valid for a wide range of *R*_*in*_(*λ*_1_, *λ*_2_), *h*_*A*_, and *h*_*B*_, provided that reasonable assumptions are made (e.g., *R*_*in*_(*λ*_1_, *λ*_2_) decreases with increasing |*λ*_2_ − *λ*_1_|, while *h*_*A*_ and *h*_*B*_ are symmetric in Δ*λ* and decrease with increasing |Δ*λ*|).

### Data acquisition and processing

Spectrometers A and B were built for visible light spectral/Fourier domain OCT systems for in vivo mouse and human retinal imaging with supercontinuum light sources (EXW-12 and EXU-3, NKT Photonics) with pulse repetition rates of 78 MHz and 156 MHz, respectively. Each spectrometer has a transmission grating (1800 l/mm @ 532 nm, Wasatch Photonics) and a complementary metal-oxide semiconductor (CMOS) line scan camera (SPL 4096–140 km, Basler) with a nominal 20 μm pixel height and a 10 μm pixel pitch. Spectrometer A (mouse) uses a 33 mm focal length reflective collimator (RC08APC-P01, Thorlabs) and a 75 mm effective focal length achromatic doublet pair focusing lens (AC508-150-A, Thorlabs), while spectrometer B (human) uses a 50.8 mm focal length reflective collimator (RC12PC-P01, Thorlabs) and a 125 mm effective focal length achromatic doublet pair focusing lens (AC508-250-A, Thorlabs). All spectral resolution characterizations in the main text were performed on spectrometer A. Our characterization and calibration approaches were validated with a 1 mW collimated laser diode at 635 nm (CPS180, Thorlabs) and a 4.5 mW collimated laser-diode-pumped laser module at 532 nm (CPS532, Thorlabs). The characterization and calibration methods utilized time courses with 32768 points acquired at a 70 kHz line rate. The spectrum intensity was maximized while avoiding saturation to increase excess noise for robust measurements. The light intensity was controlled by a variable neutral density filter in the reference arm, and time courses were acquired with the sample arm covered. For demonstration of the cross-calibration method, spectrometer A (mouse) was used to calibrate spectrometer B (human) using the supercontinuum light source (EXW-12, NKT Photonics).

### Mouse retinal imaging with visible light OCT

A free-space visible light spectral/Fourier domain OCT system^20^ was used for in vivo retinal imaging of one- to eighteen-month-old mice, as approved by our Institutional Animal Care and Use Committee (IACUC). Experiments were performed on four pigmented mice (C57BL/6J, The Jackson Laboratory) and two albino mice (BALB/cJ, The Jackson Laboratory). In addition to the improved spectrometer, several additional improvements were incorporated relative to a previous report^20^. We replaced the 50/50 beamsplitter with a 90/10 beamsplitter (BS028, Thorlabs) and added a polarization controller (FPC-3, Fiber Control) to the fiber connected to the spectrometer. We also broadened the bandwidth by utilizing all 4096 sensor pixels instead of the previous 3072 pixel configuration^20^. The full spectral width used for imaging was 259 nm, and the axial resolution was 1.0 μm in tissue. Retinal imaging was performed with a 300 μW power on the cornea with a 30 kHz line rate. Eight repeated volumetric datasets with 512 a-lines and 128 b-scans each over 17.5 s were acquired over a 1 mm range along the fast axis, with a total slow axis offset of 0.12 mm range for speckle reduction. The raw fringes were processed with linear wavenumber resampling, spatially dependent dispersion compensation^20^, spectral shaping, Fourier transformation, and axial motion correction. Images were averaged prior to display.

## Supplementary information

Supplementary material
