# Supplementary material for: Incoherent excess noise spectrally encodes broadband light sources
Source: Light Sci Appl. 2020 Oct 6;9:172. doi: 10.1038/s41377-020-00404-6 (PMC7538909; doi:10.1038/s41377-020-00404-6)
Supplement: Supplementary file 1 — Supplementary material [file 41377_2020_404_MOESM1_ESM.pdf]

Supplementary information

Incoherent excess noise spectrally encodes broadband light sources

Aaron M. Kho[1], Tingwei Zhang[1], Jun Zhu[1], Conrad W. Merkle[1], Vivek J. Srinivasan[1,2]

[1]Department of Biomedical Engineering, University of California Davis, Davis, CA, USA 95616
[2]Department of Ophthalmology and Vision Science, University of California Davis School of Medicine, Sacramento, California, USA

1. Spectral resolution theoretical limit and impulse response shape

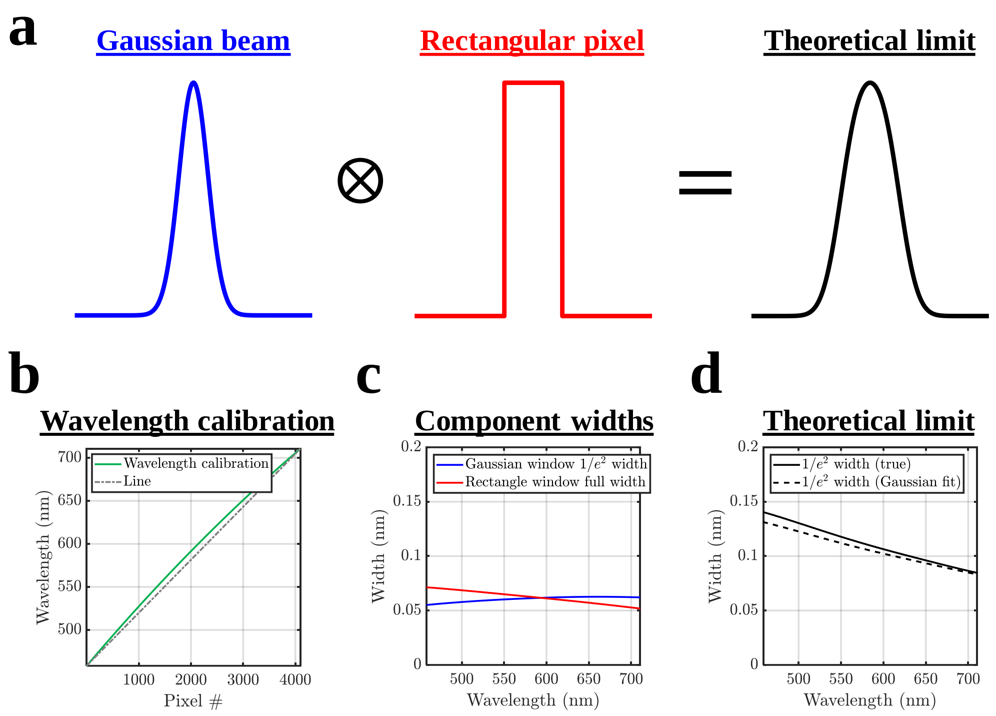

Fig. S1. Theoretical limit of spectral resolution. a The theoretical impulse response that yields the spectral resolution limit (black) is obtained through the local convolution, denoted by "⊗", of the transverse Gaussian beam on the camera sensor (blue) with the rectangular width of the sensor pixel (red). **b** Wavelength versus sensor pixel. Note that the wavelength is not linear with pixel number. **c** The spectral widths of the Gaussian beam (blue) and the rectangular pixel (red) for every wavelength in the spectrum. **d** The true width of the theoretical impulse response limit is similar to the fitted Gaussian width for all wavelengths.

Not accounting for aberrations and pixel cross-talk, we assume that the theoretical spectral resolution of a spectrometer is given by the local convolution of the transverse Gaussian beam on the sensor with the rectangular pixel[1,2] (Fig. S1a). Given the estimated mode field diameter at all wavelengths and the focal lengths of the collimator and focusing lens in Spectrometer A, we calculate the wavelength-dependent Gaussian spot size on the sensor. Both the Gaussian and rectangular window widths (Fig. S1c) are converted to spectral widths, according to our

wavelength calibration[3] (Fig. S1b), and are locally convolved to obtain the spectral resolution theoretical limit (Fig. S1d). This simulation does not account for chromatic aberrations or the change in refractive index of the core and cladding with wavelength, which changes the numerical aperture. The $\frac{1}{e^2}$ width of a fitted Gaussian is comparable to the true $\frac{1}{e^2}$ width of the theoretical limit (Fig. S1d). Given the small error of the width determined by Gaussian fitting, we conclude that Gaussian fitting can correctly infer the spectral resolution from the impulse response, and by extension, the excess noise autocorrelation matrix.

2. Ray tracing to predict spectrometer performance

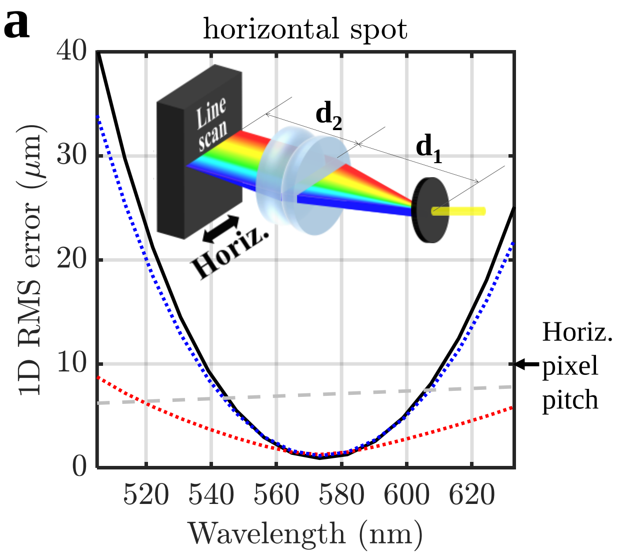

Fig. S2. Horizontal (a) and vertical (b) one-dimensional (1D) root-mean-squared (RMS) spot sizes at the sensor, based on ray tracing, explain the performance improvement observed in Fig. S12a. Ray tracing was performed in OpticStudio (Zemax, LLC) with variable d_1, d_2, and sensor horizontal tilt, with a 9.5 mm pupil to optimize either the vertical direction (black line) or the horizontal direction (dotted red line). A standard back focal plane configuration is also shown for comparison (dotted blue line). Optimizing the vertical direction yields a poor horizontal 1D RMS error (black in a), hence poor spectral resolution, towards the edges of the spectrum. On the other hand, optimizing the horizontal direction improves the horizontal 1D RMS error (dotted red line in a) to near the 1D diffraction limit (dashed gray line in a) and below the horizontal pixel pitch (arrow in a), without degrading the vertical 1D RMS error (dotted red line in b) significantly relative to the vertical pixel size (arrow in b). Even accounting for diffraction effects (dashed gray line in b), we suggest that horizontal optimization does not incur significant loss of light compared to vertical optimization, as both the vertical 1D RMS error and diffraction limit remain less than half the vertical pixel size (b). Optimal distances in simulation were d_1 = 139.9 mm and d_2 = 118.8 mm for vertical optimization (black), d_1 = 72.0 mm and d_2 = 118.8 mm for horizontal optimization (dotted red), and d_1 = 118.8 mm and d_2 = 118.8 mm for the back focal plane configuration (dotted blue). The diffraction limit was estimated from the spectrometer magnification of 2.4, assuming a fibre mode field diameter of 3.5 microns at 488 nm.

We performed ray tracing (Fig. S2) of the spectrometer later characterized in Supplementary Note 10 (spectrometer B) to better understand the origins of the experimentally observed improvements in performance. Simulations were performed to optimize either the vertical direction, and thus intensity (black line), or the horizontal direction, and thus spectral resolution (dotted red line). Individually optimized configurations were compared against the back focal plane configuration, where the grating is placed in the back focal plane of the lens (dotted blue line).

Optimization of the vertical direction resulted in poor performance along the horizontal direction (Fig. S2a), as measured by the one-dimensional (1D) root-mean-squared (RMS) error. On the other hand, it was possible to significantly improve the horizontal RMS error (Fig. S2a) without degrading the vertical RMS error (Fig. S2b) enough to significantly impact performance. This is due in part to the vertical pixel size of 20 microns (arrow in Fig. S2b), which is tolerant to vertical aberrations. These simulations justify our experimental approach of choosing the lens-to-grating distance to optimize the spectral resolution (horizontal direction), as this configuration also achieves near-optimal intensity.

Note that while spectrometer optimization driven by ray tracing provides insight into the improved performance, simulations cannot substitute for experimental measurements of spectral resolution. For instance: 1) Tolerances for the fibre mode field diameter and numerical aperture at visible wavelengths are on the order of 15%, leading experimental uncertainty. 2) Pixel cross-talk, which increases the effective sensor pixel size, is wavelength dependent and challenging to measure and model. 3) Manufacturing tolerances necessitate some form of mechanical adjustment, particularly for homebuilt spectrometers.

3. Autocorrelation matrix estimation and normalization

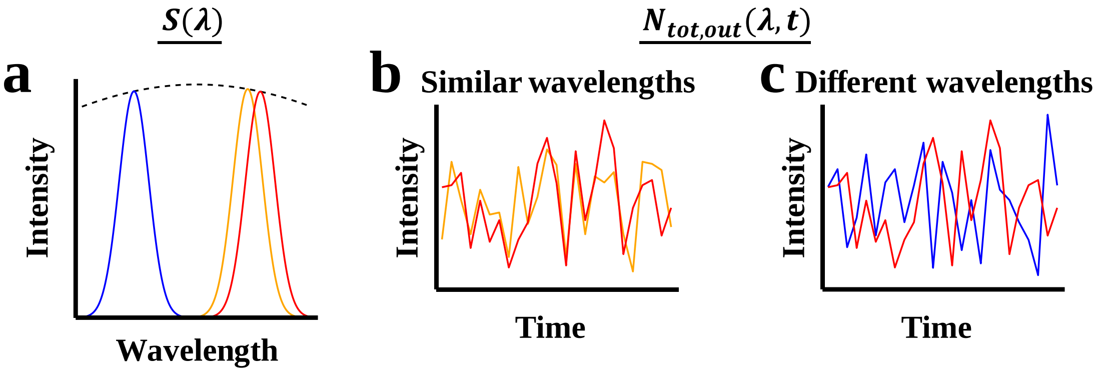

Fig. S3. Excess noise spectrally encodes broadband light. a Three narrowband channels, e.g. spectrometer pixels, are represented as individual colors within a broadband spectrum (dotted line). **b** Since the red and orange channels measure overlapping wavelengths (**a**), their excess noise fluctuations are similar, leading to higher correlation. **c** By comparison, since the red and blue channels do not measure overlapping wavelengths (**a**), their excess noise fluctuations are dissimilar, leading to lower correlation. This simple principle forms the basis for the proposed methods of spectrometer characterization and cross-calibration.

Our measured spectrum comprises spectral channels at every wavelength, λ, each with a corresponding intensity, $S(\lambda)$ (Fig. S3a). The noise values (Fig. S3b,c) at each time point, t, measured by the spectrometer after mean subtraction, $N_{tot,out}(\lambda,t)$, are represented by an X by N matrix, with X being the number of sensor pixels, or wavelengths, and N being the total number of lines in time. The autocorrelation of $N_{tot,out}(\lambda,t)$ yields the X by X symmetric total noise autocorrelation matrix, $R_{tot}(\lambda_1,\lambda_2)=\langle N_{tot,out}(\lambda_1,t)N_{tot,out}(\lambda_2,t)\rangle_t$, where $\langle\cdot\rangle_t$ denotes a time average. Since

total noise includes independent contributions from detector noise (comprising dark noise and read noise), shot noise, and excess noise, we estimated the excess noise autocorrelation matrix, $R_{out}(\lambda_1,\lambda_2)$, described in the Methods section, by subtraction of the detector noise autocorrelation matrix, $R_{detector}(\lambda_1,\lambda_2)$ and the shot noise autocorrelation matrix, $R_{shot}(\lambda_1,\lambda_2)$:

$$R_{out}(\lambda_1,\lambda_2) = R_{tot}(\lambda_1,\lambda_2) - R_{detector}(\lambda_1,\lambda_2) - R_{shot}(\lambda_1,\lambda_2) \tag{1}$$

$R_{detector}(\lambda_1,\lambda_2)$ is estimated similarly to $R_{tot}(\lambda_1,\lambda_2)$, albeit from a dataset acquired with no light on the detector. $R_{shot}(\lambda_1,\lambda_2)$ is a diagonal matrix with the shot noise variance along the diagonal. Note that while sometimes relative intensity noise (RIN) and excess noise are used interchangeably[4,5], this usage is not universal and we refrain from discussing RIN. Since shot noise follows a Poisson distribution, the variance in a given pixel can be estimated from the source spectrum gray level (DN), given by $S_{out}(\lambda)$, in that pixel given, the Full Well Capacity (FWC) and bit depth (BD):

$$R_{shot}(\lambda,\lambda) = S_{out}(\lambda)\left(\frac{FWC}{2^{BD}}\right)\left(\frac{2^{BD}}{FWC}\right)^2 = S_{out}(\lambda)\left(\frac{2^{BD}}{FWC}\right) \tag{2}$$

When the full-well capacity of the camera was not precisely known, it was estimated by performing a quadratic polynomial fit of the pixel noise versus source spectrum gray level[6]. After normalization to the product of the excess noise standard deviations in the corresponding pixels, we obtain the experimental normalized excess noise autocorrelation matrix:

$$r_{out}(\lambda_1,\lambda_2) = \frac{R_{out}(\lambda_1,\lambda_2)}{\sqrt{R_{out}(\lambda_1,\lambda_1)R_{out}(\lambda_2,\lambda_2)}} \tag{3}$$

where the diagonals of the autocorrelation matrix, $R_{out}(\lambda,\lambda)$, represent the variance. An alternative normalization to the source spectrum gray level at each wavelength, $S_{out}(\lambda)$, yields the matrix, $r_{out}^{coeff}(\lambda_1,\lambda_2)$, in terms of the relative magnitude of excess noise fluctuations. This normalization enables direct comparison of excess noise correlations between different light sources or spectrometers. For this normalization, the diagonal is the excess noise coefficient, or the variance of intensity fluctuations relative to the mean intensity squared.

$$r_{out}^{coeff}(\lambda_1,\lambda_2) = \frac{R_{out}(\lambda_1,\lambda_2)}{S_{out}(\lambda_1)S_{out}(\lambda_2)} \tag{4}$$

Broadening of the quasi-diagonal of $r_{out}(\lambda_1,\lambda_2)$ was quantified by the full-width-at-half-maximum (FWHM) of a fitted Gaussian function centred on the diagonal (Fig.

S4). The FWHM of $r_{out}(\lambda_1,\lambda_2)$, assumed to be equal to the FWHM of $\mathrm{R}_{out}(\lambda_1,\lambda_2)$, in units of pixels was converted to FWHM spectral resolution, $FWHM_h(\lambda)$, based on Eq. (12) in the main manuscript and our pixel-to-wavelength calibration[3] (Fig. S1b).

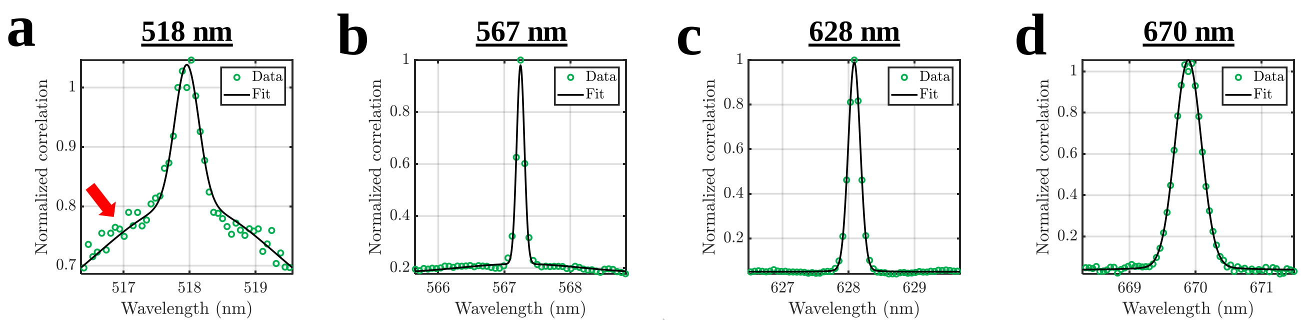

Fig. S4. Autocorrelation matrix quasi-diagonal fitting. Two Gaussian fitting of the autocorrelation matrix along the diagonal at wavelengths 518 nm (**a**), 567 nm (**b**), 628 nm (**c**), and 670 nm (**d**). The red arrow indicates the appearance of the broader Gaussian pedestal at shorter wavelengths that is not informative about the spectrometer spectral resolution.

Some spectral ranges of the supercontinuum contained an additional excess noise component, evidently not related to spectral resolution. This additional component appeared as a broader Gaussian pedestal (Fig. S4a, red arrow) in the excess noise autocorrelation function, with a correlation length of 2-3 nm. Within our measured bandwidth, this broader excess noise component was most significant below 555 nm. This component is likely due to intrinsic noise correlations across broader spectral ranges of the light source, which violate the white noise assumption[7]. To avoid confounding our estimates of spectral resolution, we fit a two Gaussian model where spectral resolution was extracted from the more informative, narrower Gaussian component.

4. Light source intrinsic spectral correlation length

Our method of spectrometer characterization, as implemented, assumes a short excess noise spectral correlation length, so that any measured spectral correlation is attributable to our spectrometer impulse response function (Eq. (9) in the main manuscript). Here, by extending the linear shift-variant system analysis in the Methods section, we assess the validity of this assumption. In particular, we experimentally place bounds on the intrinsic spectral correlation length of the light sources.

Extending on the analysis in the main text, we relax the assumption in Eq. (5) in the main manuscript that $R_{in}(\lambda_1,\lambda_2)$ is a delta function. Thus, we start from Eq. (4) in the main manuscript:

$$R_{out}(\lambda_1,\lambda_2) = \iint R_{in}(\Lambda_1,\Lambda_2)\, h(\Lambda_1,\lambda_1-\Lambda_1)\, d\Lambda_1\, h(\Lambda_2,\lambda_2-\Lambda_2)\, d\Lambda_2 \quad (5)$$

We further assume that the intrinsic correlation function (including the wavelength-dependent variance and intrinsic spectral correlation length) is slowly varying on the scale of the spectral resolution (This is less restrictive than the main text, where we assume the intrinsic spectral correlation length is much smaller than the spectral resolution). Our assumption leads to the natural reparameterization $R_{in}^{'}\left(\lambda_{avg},\Delta\lambda\right)=R_{in}\left(\lambda_{avg}-\frac{\Delta\lambda}{2},\lambda_{avg}+\frac{\Delta\lambda}{2}\right)$, where $\lambda_{avg}=\frac{\lambda_1+\lambda_2}{2}$ and $\Delta\lambda=\lambda_2-\lambda_1$, where $R_{in}^{'}$ is slowly varying in the first argument, and symmetric in the second argument, yielding

$$R_{out}\left(\lambda_1,\lambda_2\right)=\iint R_{in}^{'}\left(\frac{\Lambda_1+\Lambda_2}{2},\Lambda_2\text{-}\Lambda_1\right)h\left(\Lambda_1,\lambda_1-\Lambda_1\right)d\Lambda_1 h\left(\Lambda_2,\lambda_2-\Lambda_2\right)d\Lambda_2 \tag{6}$$

We then make the substitutions $\Lambda_1'=\lambda_1-\Lambda_1$ and $\Lambda_2'=\lambda_2-\Lambda_2$ to yield

$$R_{out}\left(\lambda_1,\lambda_2\right)=\iint R_{in}^{'}\left[\frac{\lambda_2+\lambda_1-\left(\Lambda_2'+\Lambda_1'\right)}{2},\lambda_2-\lambda_1-\left(\Lambda_2'-\Lambda_1'\right)\right]h\left(\lambda_1-\Lambda_1',\Lambda_1'\right)d\Lambda_1'h\left(\lambda_2-\Lambda_2',\Lambda_2'\right)d\Lambda_2' \tag{7}$$

As $R_{in}^{'}$ and h are both assumed to be slowly varying in their first arguments compared to their second arguments, we can approximate that

$$R_{out}\left(\lambda_1,\lambda_2\right)=R_{in}^{'}\left(\lambda_{avg},\Delta\lambda\right)\otimes\left[h\left(\lambda_{avg},\Delta\lambda\right)\star h\left(\lambda_{avg},\Delta\lambda\right)\right] \tag{8}$$

where $\star$ and $\otimes$ denote cross-correlation and convolution, respectively, with respect to $\Delta\lambda$. This suggests the natural reparameterization, $R_{out}^{'}\left(\lambda_{avg},\Delta\lambda\right)=R_{out}\left(\lambda_{avg}-\frac{\Delta\lambda}{2},\lambda_{avg}+\frac{\Delta\lambda}{2}\right)$. The term in square brackets represents the ideal excess noise correlation function in the absence of intrinsic spectral correlations in the light source. Thus, according to Eq. (8), the effect of intrinsic source excess noise correlations can be modelled as an additional convolution in $\Delta\lambda$. As $\Delta\lambda$ is related to the distance from the diagonal, the convolution implies a broadening of the quasi-diagonal. At the same time, Eq. (8) suggests a way to estimate intrinsic source correlations, if present. In particular, the width of $h\star h$ may be estimated accurately from the interferometry method, which does not require uncorrelated excess noise. At the same time $R_{out}^{'}\left(\lambda_{avg},\Delta\lambda\right)$ is the excess noise autocorrelation matrix. We empirically find that both h and $R_{out}^{'}$ are nearly Gaussian in their second arguments. If all relevant functions are Gaussian, then

$$\left[FWHM_{R_{out}^{'}}\left(\lambda_{avg}\right)\right]^2=\left[FWHM_{R_{in}^{'}}\left(\lambda_{avg}\right)\right]^2+2\left[FWHM_h\left(\lambda_{avg}\right)\right]^2 \tag{9}$$

Thus, the squared width of $R_{in}^{'}$ can be estimated from a deconvolution:

$$\left[FWHM_{R_{in}^{'}}\left(\lambda_{avg}\right)\right]^2=\left[FWHM_{R_{out}^{'}}\left(\lambda_{avg}\right)\right]^2-2\left[FWHM_h\left(\lambda_{avg}\right)\right]^2 \tag{10}$$

Here, $\left[FWHM_{R'_{out}}\left(\lambda_{avg}\right)\right]^2$ is determined from Gaussian fitting of the excess noise autocorrelation matrix and $\left[FWHM_h\left(\lambda_{avg}\right)\right]^2$ is determined from Gaussian fitting of the coherence rolloff, and Eq. (11) in the main manuscript.

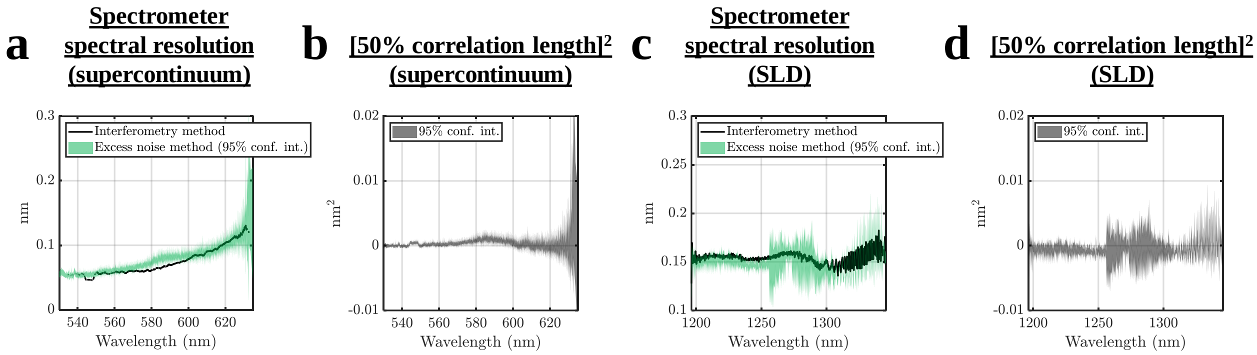

Fig. S5. Intrinsic light source spectral correlations. Spectral resolution of visible light spectrometer B (Fig. S12b) using a supercontinuum source (**a)** and commercial 1300 nm spectrometer (Fig. S10a-b) using an SLD (**c**). Systematic overestimation by the excess noise characterization method relative to interferometry would indicate intrinsic light source correlations. To investigate this possibility, applying the deconvolution in Eq. (10), the light source intrinsic spectral correlation length squared is shown for both the supercontinuum source (**b**) and the SLD (**d**). Note that the correlation length squared is determined, as sometimes the right hand side of Eq. (10) is negative. Further investigations of this effect are warranted, particularly for supercontinuum sources, using higher resolution spectrometers and longer integration times.

Spectral resolution estimates are reproduced with supercontinuum (Fig. S5a) and SLD light sources (Fig. S5c) using both interferometry, i.e. $FWHM_h$, and the proposed excess noise method, i.e. $FWHM_{R'_{out}}/\sqrt{2}$, including confidence intervals. Based on Eq. (10), we find confidence intervals for the intrinsic 50% correlation length squared, determined as $\left[FWHM_{R'_{in}}/2\right]^2$ (Fig. S5b,d). Accounting for confidence intervals, there are no clear deviations from zero for either light source. For more precise investigation of the intrinsic spectral correlation length, particularly for the supercontinuum source, further investigation using higher resolution spectrometers such as those with virtually imaged phase arrays, relaxation of the Gaussian assumption, and longer integration times, are warranted.

While the analysis in the main manuscript, which neglected intrinsic light source correlations, appears valid for spectrometers and sources investigated herein, three additional comments are in order. First, for the supercontinuum source, we were able to exclude correlations on the few nanometer scale by fitting a two Gaussian model (see Fig. S4 and discussion at the end of Supplementary Note 3). Second, in applications where intrinsic source correlations (i.e. R'_{in}) are present and known *a priori*, they can be incorporated into the analysis, as per Eq. (8), to improve accuracy. Third, there are theoretical arguments supporting the incoherence of excess noise from pure spontaneous emission.

5. Origins of incoherent excess noise

Beyond the experimental data presented in this work, theoretical arguments support that pure spontaneous emission sources exhibit incoherent excess noise, uncorrelated at optical frequencies spaced much greater than the inverse integration time. The predominant expression for excess noise in the literature[4,5,8] for a single mode, thermal source with Gaussian field statistics assumes excess intensity fluctuations result from the addition of independent temporal cells. The cell number (N) is given by the ratio of the detector integration time to the coherence time[9], i.e. $N = T/\tau_c$, leading to $1/N$ scaling of the excess noise coefficient. Equivalently, the number of independent cells is proportional to the ratio of the optical linewidth to the detection bandwidth[10]. If the effective optical linewidth is equated to the spectrometer spectral resolution, this theory implies negligible intrinsic correlation length, i.e. incoherent excess noise. How well do amplified spontaneous emission sources obey the incoherent excess noise assumption, particularly in the presence of gain saturation that could introduce correlations between wavelengths? While further work is needed, experimental measurements[5] suggest that, with an empirical factor to account for noise suppression, the predicted inverse linewidth scaling of excess noise is indeed observed for superluminescent diodes (SLDs).

For supercontinuum sources, the intrinsic correlation length is complex and critically depends on the nonlinear processes involved in spectral broadening[11-13]. For broad supercontinuum generation in a photonic crystal fibre, the degree to which modulation instability is seeded coherently (e.g. by a short pulse) versus by noise on the input pulse is a key determinant of final coherence[11]. Notably, in a low noise 1700 nm source based on self-phase modulation, incoherent excess noise was not detected (Fig. S8f). However, for photonic crystal fibre-based broadband supercontinuua, a sufficiently incoherent excess noise component was inferred both at 1600-1800 nm (Fig. S8d,e) and at visible wavelengths (e.g., Fig. S4 and Fig. S9a). A fitting procedure to exclude broader intrinsic spectral correlations and isolate this component (Fig. S4 and Supplementary Note 3) enabled accurate determination of spectral resolution.

6. Spectral resolution with narrowband laser and interferometry methods

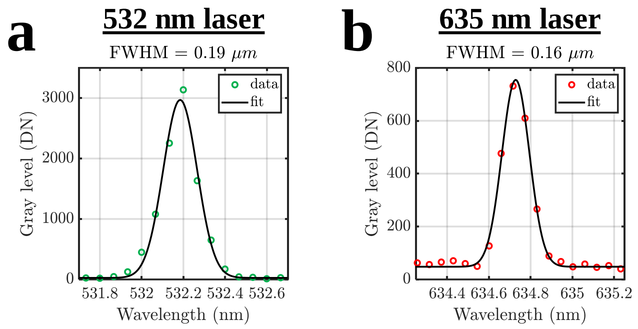

Fig. S6. Narrowband laser method for spectral resolution. Gaussian fitting of the measured green (~532 nm) (**a**) and red (~635 nm) (**b**) laser spectra to obtain the spectral resolution.

The spectral resolution determined from the proposed excess noise method was validated against two well-accepted methods (Fig. 2b): narrowband lasers and broadband interferometry[1,2,14]. For the narrowband laser method, if the laser linewidth is significantly narrower than the spectral resolution, the FWHM of the spectral intensity distribution measured by the spectrometer is the spectral resolution (Fig. S6). Note that even if the laser width is comparable to the spectral resolution, the spectral resolution is still obtainable if the laser lineshape is known. Here, the lineshape was assumed to approximate a delta function.

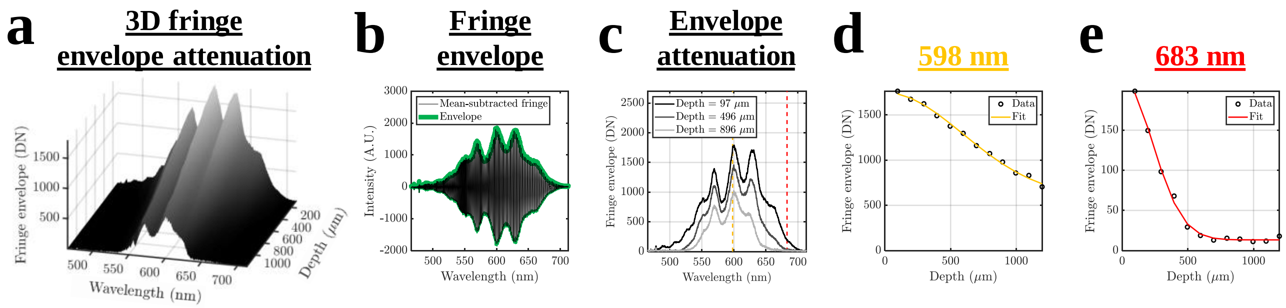

Fig. S7. Interferometry method for spectral resolution. a Interference fringe envelope versus wavelength and depth (path length mismatch divided by two). To highlight the effects of wavelength-variant spectral resolution, the original, sub-optimal configuration of spectrometer A (Fig. 3a) was used. **b** The interference fringe and its envelope at a depth of 97 μm. **c** Fringe envelope, as a function of wavelength, is shown at various depths with the colored dotted lines showing wavelengths in **d** and **e**. Fringe envelope attenuation with depth is shown at λ = 598 nm (**d**) and λ = 683 nm (**e**).

For the broadband interferometry method, the attenuation of the interference fringe envelope with increasing spectral oscillation rate (path length) is caused by the finite spectral distribution measured by each pixel. Multiple datasets at different path length mismatches were acquired to obtain the attenuation of the fringe envelope with depth (path length divided by two), for each pixel or wavelength in the spectrum (Fig. S7a-c). For each pixel/wavelength (Fig. S7d-e), we fit a Gaussian function to the interference fringe attenuation versus depth, whose FWHM width, $\Delta z(\lambda)$, is the axial resolution for optical coherence tomography (OCT), at that

corresponding pixel/wavelength. Since the spectral resolution can also be viewed as the spectral bandwidth at a given pixel, we can derive the spectral resolution from $\Delta z(\lambda)$ using the OCT axial resolution equation[15]:

$$FWHM_h(\lambda) = \frac{2ln2}{\pi}\frac{\lambda^2}{\Delta z(\lambda)} \tag{11}$$

where $FWHM_h(\lambda)$ is the spectral resolution and λ is the centre wavelength at the given pixel.

7. Feasibility with other light sources

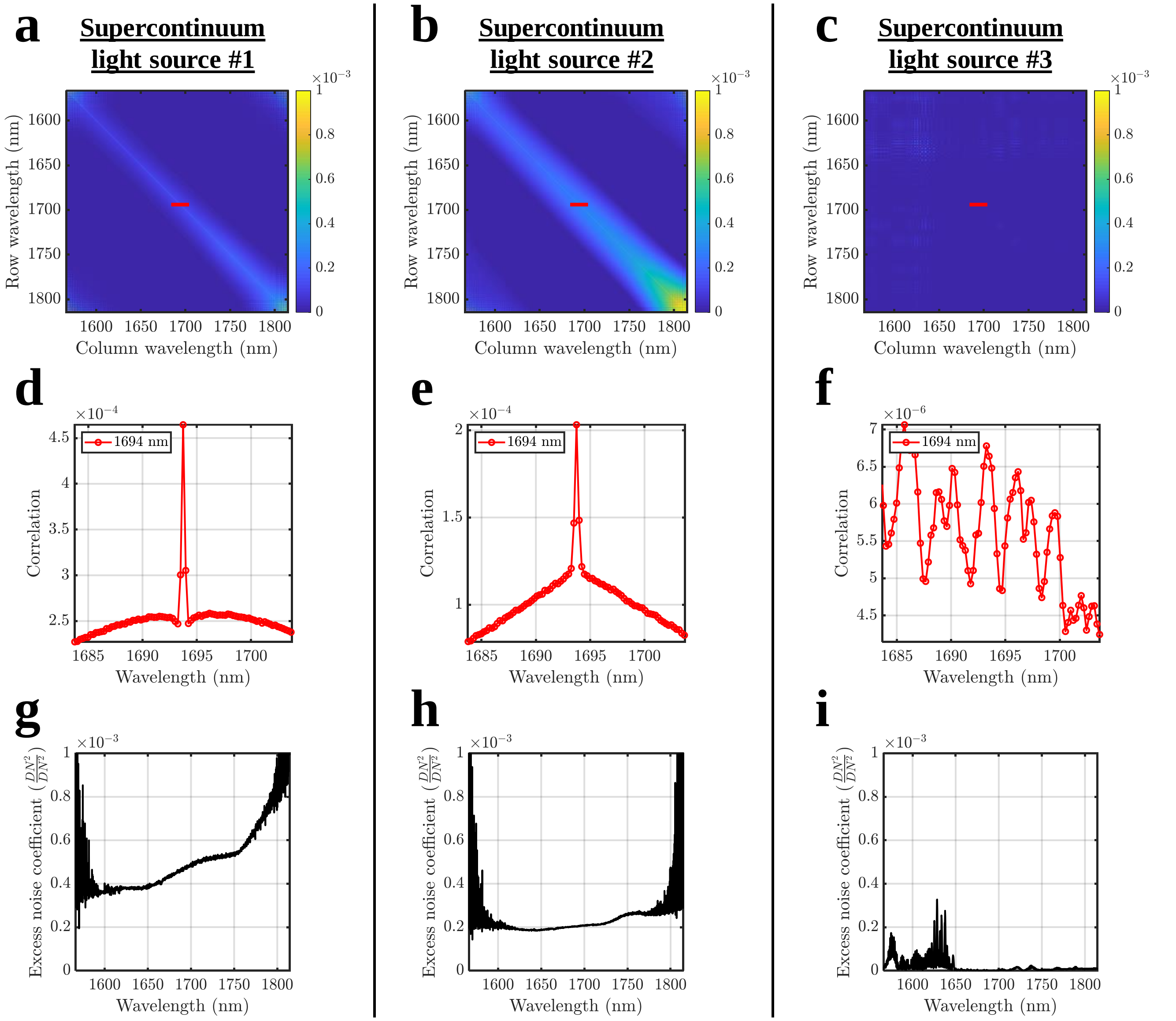

Fig. S8. Feasibility of spectrometer characterization method with different supercontinuum light sources. a-c Normalized excess noise autocorrelation matrices in coefficient form [Eq. (4)] for supercontinuum sources #1 (**a**), #2 (**b**), and #3 (**c**). Red lines in **a-c** represent the locations

of **d-f**. The narrow peaks in zooms around the diagonal near 1694 nm (**d,e**), which are informative about the spectral resolution, are not apparent in **f**. Respective excess noise coefficients (diagonals of **a-c**), plotted in **g-i**, show that source #3 possesses the least excess noise.

Our characterization approach was applied to a spectrometer centred around 1700 nm (1567-1815 nm spectral range), to investigate the performance of supercontinuum light sources from different manufacturers with different excess noise levels. Light source #1 and light source #2 (EXW-12, NKT Photonics) were broadband supercontinua based on photonic crystal fibres with repetition rates of 40 MHz and 78 MHz, respectively. Light source # 3 had a repetition rate of 50 MHz, but produced a narrower spectrum from self-phase modulation.

The characterization method was applied to time courses with 46900 points acquired at a 47 kHz line rate. Spectrometer calibration was deemed possible with a quasi-diagonal excess noise component (Fig. S8a,b) that correctly predicted the spectral resolution determined by broadband interferometry. For each source, we also quantified the excess noise coefficient as described previously described (Fig. S8g-i)[6]. More excess noise appears to give more robust measurements (Fig. S8a,b,d,e,g,h). Importantly, the characterization failed with the supercontinuum light source based on pure self-phase modulation, which also had significantly less excess noise (Fig. S8c,f,i). Self-phase modulation may also lead to correlated fluctuations at different wavelengths, invalidating the white noise assumption.

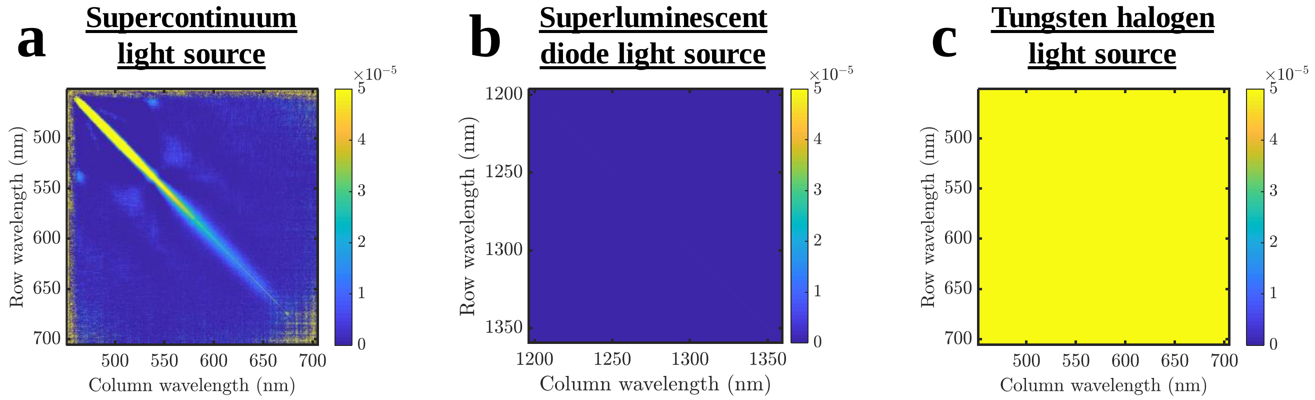

Fig. S9. Feasibility of spectrometer characterization method with non-supercontinuum light sources. Normalized excess noise autocorrelation matrices in coefficient form [Eq. (4)] for a supercontinuum source (**a**), superluminescent diode (SLD) (**b**), and a tungsten halogen light source (**c**) show that the significant excess noise correlations along the quasi-diagonal are not apparent in all broadband sources.

Furthermore, we assessed the feasibility of applying our approach with non-supercontinuum light sources: a superluminescent diode (SLD1325, Thorlabs) (Fig. S9b) and a tungsten halogen light source (JCR21V-150W, Ushio) (Fig. S9c). Autocorrelation matrices were normalized according to Eq. (4) and compared to those of the original supercontinuum light source used in this study (EXW-12, NKT Photonics) (Fig. S9a). Correlations were observed with the superluminscent diode light source, though excess noise levels were very low (Fig. S9b). Further investigation (Supplementary Note 8) later revealed that indeed, excess noise

characterization was possible with the SLD source. The tungsten halogen source, though having large excess noise, likely related to technical noise, did not clearly show excess noise correlations along the quasi-diagonal, which are required for characterization (Fig. S9c).

8. Excess noise in a superluminescent diode (SLD) can also characterize spectrometers

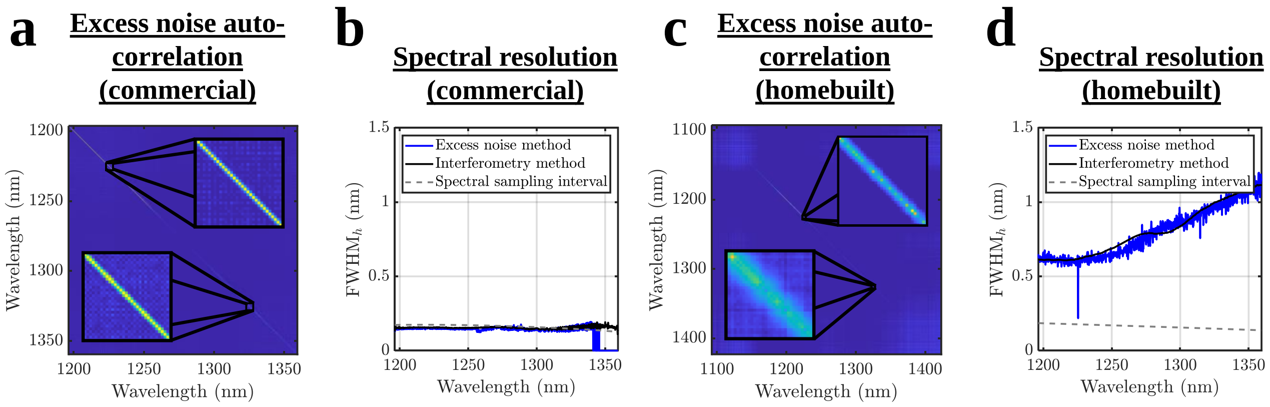

Fig. S10. Spectrometer characterization with a superluminescent diode (SLD) source. Excess noise autocorrelation matrices (**a,c**) and estimated spectral resolutions (**b,d**), employing an SLD source with both a commercial spectrometer (**a,b**) and a homebuilt spectrometer (**c,d**), the latter with a coarser spectral resolution. Insets in **a,c** are zooms of the respective quasi-diagonals from 1223-1228 nm (upper right) and from 1323-1328 nm (lower left). Although the homebuilt spectrometer (**d**) has a larger spectral range, the spectral resolution is only shown across the spectral range of the commercial spectrometer (**b**) to facilitate comparison.

Superluminescent diodes (SLDs) are less expensive and exhibit lower excess noise than supercontinuum sources, making them the light source of choice for OCT and many sensing applications. Thus, we investigated the feasibility of employing an SLD for spectrometer characterization, with an extended integration time of 0.72 seconds (compared to the 0.47 seconds used for visible light supercontinuum sources). Spectral resolution measurements were first performed on a 91 kHz line rate spectrometer inside a commercial OCT system (TELESTO-II, Thorlabs), using the built-in SLD light source (SLD1325, Thorlabs). The non-uniform spectral shape of the commercial SLD prevents simultaneously operating near the full well capacity of each pixel, which is desirable to maximize contribution of excess noise relative to other noise sources. Thus, we acquired multiple time courses with different levels of attenuation, to ensure the existence of a time course for each pixel with counts just below the full well capacity. No other measures were taken to enhance excess noise. For this spectrometer, although the spectral resolution was comparable to the spectral sampling interval (Fig. S10.b), leading to a narrow quasi-diagonal with decorrelation within two pixels from the diagonal (Fig. S10.a insets), spectral resolution measurements are nonetheless possible, showing (Fig. S10.b) good agreement with the gold standard interferometry method.

We next performed measurements on an auxiliary homebuilt spectrometer, employing the same SLD light source. This homebuilt spectrometer had a spatially varying spectral resolution that greatly exceeded the spectral sampling interval (Fig. S10.d). Briefly, the light entering the spectrometer was collimated with a 40 mm achromatic doublet (AC254-040-C, Thorlabs). A volume transmission grating (1145 lines per millimeter, Wasatch Photonics) dispersed the light, which was then focused onto a 147 kHz line scan camera (GL2048R, Sensors Unlimited) using an achromatic doublet pair with an effective focal length of 40 mm (AC300-080-C, Thorlabs). As the spectral resolution of this spectrometer was coarse relative to the spectral sampling interval, the quasi-diagonal of the excess noise correlation matrix was likewise broad (Fig. S10.c). The coarse spectral resolution was quantitatively confirmed by the interferometry method (Fig. S10.d). Since the spectral range of the homebuilt spectrometer was wider than that of the SLD light source, and neither method could characterize spectral resolution at the edges of the spectrometer range, the full spectrometer range is not shown in Fig. S10.d. In summary, excess noise characterization agreed with interferometry across two spectral resolution ranges (Fig. S10.b,d), supporting the potential to perform spectrometer calibration with excess noise of an SLD source.

Notably, despite the extended integration time, spectrometer characterization results with the SLD were noisy, particularly near the edges of the light source spectrum. We suggest that although the low noise of superluminescent diodes is an asset in OCT, reduced excess noise may be detrimental to our method of characterization, which is most efficient in the regime where excess noise dominates shot noise and detector noise. To this end, strategies such as reducing gain saturation by driving the SLD at a lower current, and reducing the exposure time while maintaining count levels near the full well capacity of each pixel, may help to improve upon the results presented here.

9. Relationship of spectral correlations to noise floor.

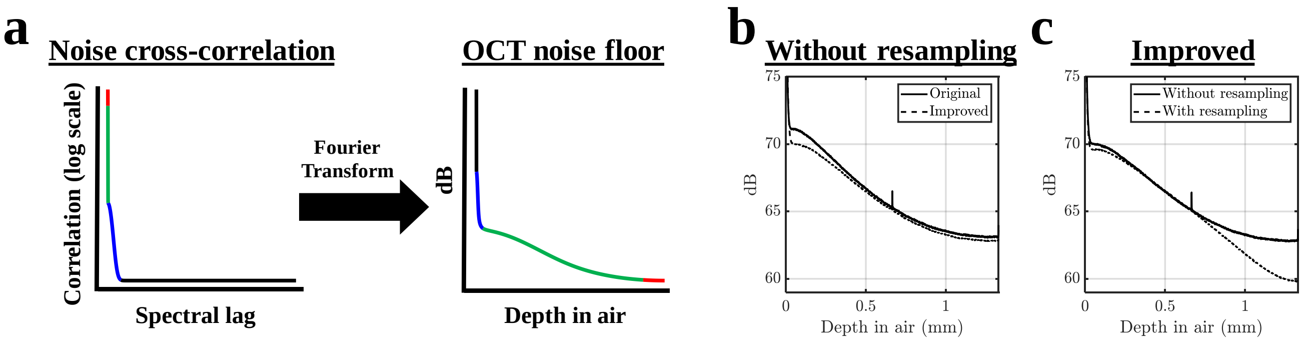

Fig. S11. Noise floor rolloff. a Diagram of noise spectral cross-correlation and its Fourier transform pair, the OCT noise floor. The red component represents shot noise, the green component represents spectral resolution, the blue component represents the broader excess noise correlations from the light source, and the black component represents spectrum fluctuations related to laser instability. **b** The improved configuration (Fig. 3b)

narrows the excess noise spectral correlations, resulting in a broader noise floor relative to the original configuration (Fig. 3a). **c** The resampling procedure broadens the noise spectral correlations digitally, resulting in a narrower noise floor with a steeper rolloff. The inverse relationship between the widths of the spectral correlations and the noise floor are understood through the Wiener-Khinchin theorem.

Spectral correlations were previously discussed in the context of supercontinuum sources and related to the rolloff of the spectral/Fourier domain OCT noise floor (the Fourier transform of the correlation function). However, in this prior work, spectral correlations, causing the noise floor reduction with depth, were attributed to the supercontinuum source[16-18]. Here we argue that the intrinsic spectral resolution of the spectrometer and the linear in wavenumber resampling procedure must both be considered in predicting the OCT noise rolloff. Notably, with an improved spectral resolution configuration (Fig. 3b), we can broaden the green component in the noise rolloff profile (Fig. S11a) to reduce the noise at short path length mismatches (Fig. S11b), without changing the light source. Also, the resampling procedure, commonly applied in OCT to ensure spectral data is linearly spaced in wavenumber, digitally induces spectral correlations and thus narrows the noise floor, reducing the noise at larger path length mismatches (Fig. S11c). Therefore, the noise rolloff, commonly observed in OCT images using supercontinuum light sources, is not only affected by the intrinsic spectral correlations of the source, but also by spectral resolution of the spectrometer and the resampling procedure.

10. Additional OCT spectrometer characterization and improvements

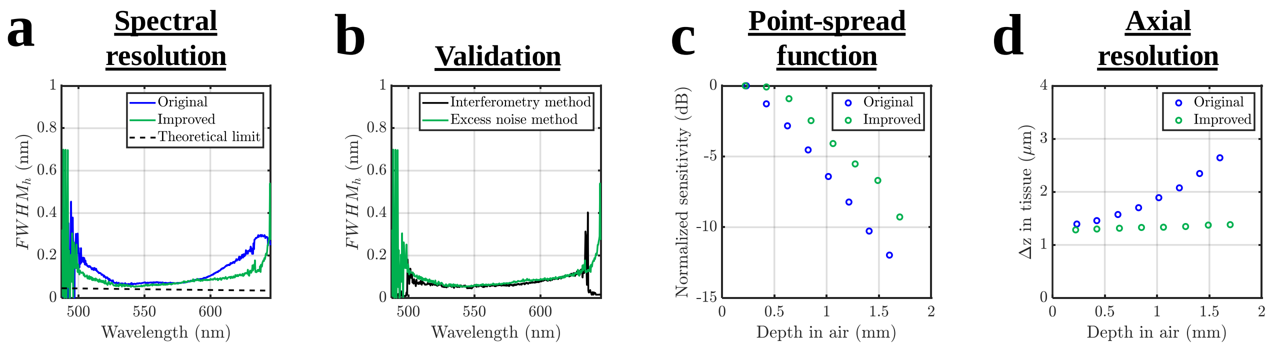

Fig. S12. Quasi-real time characterization improves human OCT spectrometer alignment. a Spectral resolution across the spectrum for the original and improved configurations of spectrometer B (analogous to Fig. 3a,b for spectrometer A), achieved using excess noise correlations for alignment. **b** Validation of excess noise method against broadband interferometry for the improved configuration. **c** OCT point-spread function rolloff. **d** Axial resolution degradation versus depth in air.

The excess noise correlation method was applied to a human visible light OCT spectrometer[19] (spectrometer B) to improve its performance by moving the focusing lens closer to the diffraction grating, as shown in Fig. 3a-b (Fig. S12a). The sensitivity rolloff improved to 6.9 dB, from 12.6 dB, and the axial resolution degradation improved to 8%, from 89%, at a 1.5 mm imaging depth in air (Fig. S12c,d). These marked improvements in spectral resolution are supported by ray tracing described in Supplementary Note 2. Having a full spectral bandwidth of 156 nm, ~0.6 times than that of spectrometer A, shown in Fig. 3 in the main manuscript,

spectrometer B achieves ~0.6 times finer spectral resolution. In addition, compared to the higher noise, lower repetition rate source used in Fig. 3 in the main manuscript, this spectrometer used a lower noise, higher repetition rate source. Still, the excess noise method showed good agreement with the broadband interferometry method (Fig. S12b). Therefore, for investigated spectrometers, our spectral resolution measurement is not limited by intrinsic spectral correlations of the supercontinuum light source, and the smallest measurable spectral resolution is less than 0.05 nm (Fig. S12b). Detailed investigation of potential intrinsic correlations is described in Supplementary Note 4.

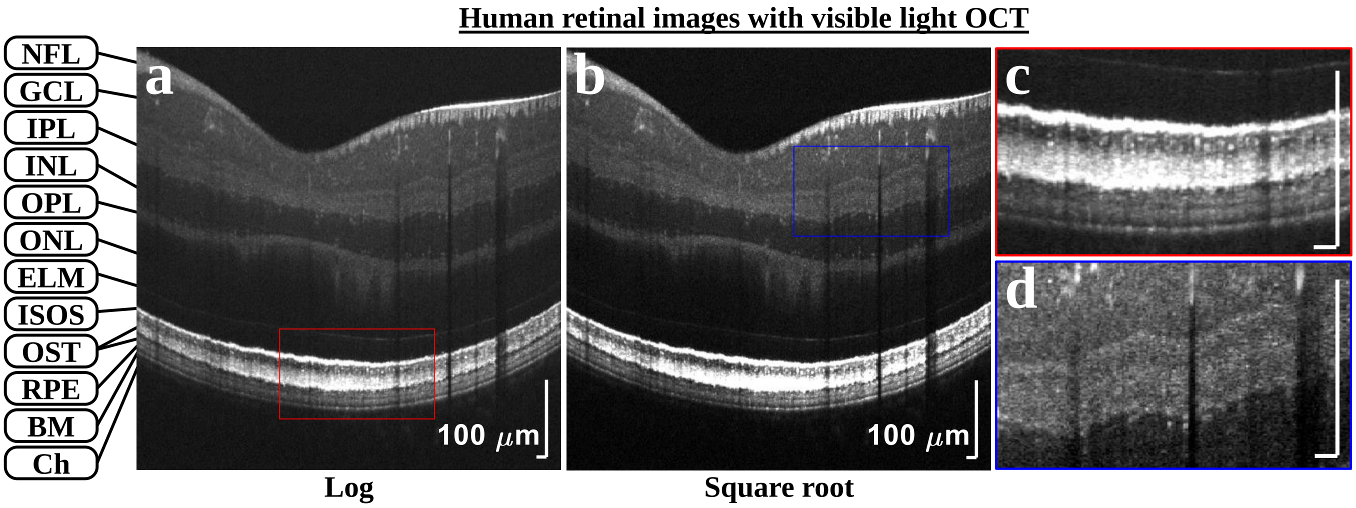

Fig. S13 Visible light OCT of the human retina. Cross-sectional images of the human retina, on log (**a**) and square root (**b**) scale, acquired by a visible light OCT system with a spectrometer aligned by excess noise correlations (NFL: Nerve Fibre Layer; GCL: Ganglion Cell Layer; IPL: Inner Plexiform layer; INL: Inner Nuclear Layer; OPL: Outer Plexiform Layer; ONL: Outer Nuclear Layer; ELM: External Limiting Membrane; ISOS: Inner Segment / Outer Segment Junction; OST: photoreceptor Outer Segment Tips; RPE: Retinal Pigment Epithelium; BM: Bruch's Membrane; Ch: Choroid).

A fibre-based visible light spectral/Fourier domain OCT system[19] with the previously described spectrometer improvements was used for *in vivo* retinal imaging of a twenty-six year old human male (Fig. S13). Retinal imaging was performed with 150 μW power on the cornea with a 30 kHz line rate. A detailed safety calculation based on ANSI Z136.1 – 2014 American National Standard for Safe Use of Lasers published by the Laser Institute of America is described previously[6,20]. All experimental procedures and protocols were reviewed and approved by the UC Davis Institutional Review Board (IRB). A volumetric dataset with 512 a-lines and 200 b-scans each was acquired over a 5 mm range along the fast axis, with a total offset along the slow axis of 0.2 mm for speckle reduction[19]. The raw fringes were processed with linear wavenumber resampling, spatially dependent dispersion compensation[21], spectral shaping[19], Fourier transformation, and transverse and axial motion correction. Images were averaged prior to display.

11. Cross-correlation matrix estimation and normalization

Spectrometer cross-calibration requires the cross-correlation of simultaneously measured noise time courses from spectrometer A, $N_{A,tot,out}(x_A,t)$, and spectrometer B, $N_{B,tot,out}(x_B,t)$, for each time point, t, and each corresponding spectrometer pixel, x_A and x_B, respectively. This yields the total noise cross-correlation matrix, $R_{AB,tot}(x_A,x_B)=\langle N_{A,tot,out}(x_A,t)\,N_{B,tot,out}(x_B,t)\rangle_t$. Unlike the autocorrelation matrix in Supplementary Note 3, $R_{AB,tot}(x_A,x_B)$ is not influenced by shot noise and detector noise, which should be uncorrelated between spectrometers. Therefore $R_{AB,tot}(x_A,x_B)$ is equal to the excess noise cross-correlation matrix, $R_{AB,out}(x_A,x_B)$, described in the Methods section. Through normalization to the product of the excess noise standard deviations in the corresponding pixel for both spectrometers, we obtain the experimental normalized excess noise cross-correlation matrix, $r_{AB,out}(x_A,x_B)$:

$$r_{AB,out}(x_A,x_B)=\frac{R_{AB,out}(x_A,x_B)}{\sqrt{R_{A,out}(x_A,x_A)\,R_{B,out}(x_B,x_B)}} \tag{12}$$

$R_{A,out}(x_A,x_A)$ and $R_{B,out}(x_B,x_B)$ are the excess noise autocorrelation matrices (see Supplementary Note 3) for each spectrometer, containing the variances for each pixel along the principal diagonal. For a given pixel in one spectrometer, the pixel in the other spectrometer with the highest correlation, given by $r_{AB,out}(x_A,x_B)$, measures similar wavelengths.

12. Quasi-real time spectral resolution calculation

A major advantage of our proposed excess noise characterization method is simple implementation. To provide continuous characterization of spectral resolution during spectrometer alignment, we incorporated a custom add-on written in Python (version 3.6) into our Labview program for control and display of our human visible light OCT system[6]. The spectral resolution was computed at 41 pixels across the sensor in 2.9 seconds, which was sufficient for quasi-real time display to facilitate spectrometer alignment. We employed this display to aid in aligning spectrometer B (Fig. S12).

Of note, it is conceivable to employ an interferometer with a mechanically adjusted path length to automate the interferometry method of determining spectral resolution. However, besides the added expense of an additional time-domain interferometer and the complexity of synchronization, the reference arm translation is limited in speed by concerns about fringe washout. Thus, we conclude that such

a simple, inexpensive, and high-speed spectral resolution measurement is impractical with the conventional interferometry approach (Fig. 1c).

Supplementary information accompanies the manuscript on the Light: Science & Applications website (http://www.nature.com/lsa)

13. References

1 Yun, S., Tearney, G., Bouma, B., Park, B. & de Boer, J. F. High-speed spectral-domain optical coherence tomography at 1.3 µm wavelength. *Optics express* **11**, 3598-3604 (2003).

2 Leitgeb, R., Hitzenberger, C. & Fercher, A. F. Performance of fourier domain vs. time domain optical coherence tomography. *Optics express* **11**, 889-894 (2003).

3 Chong, S. P., Merkle, C. W., Leahy, C., Radhakrishnan, H. & Srinivasan, V. J. Quantitative microvascular hemoglobin mapping using visible light spectroscopic Optical Coherence Tomography. *Biomedical optics express* **6**, 1429-1450 (2015).

4 De Boer, J. F. *et al.* Improved signal-to-noise ratio in spectral-domain compared with time-domain optical coherence tomography. *Optics letters* **28**, 2067-2069 (2003).

5 Shin, S., Sharma, U., Tu, H., Jung, W. & Boppart, S. A. Characterization and analysis of relative intensity noise in broadband optical sources for optical coherence tomography. *IEEE Photonics Technology Letters* **22**, 1057-1059 (2010).

6 Chong, S. P., Bernucci, M., Radhakrishnan, H. & Srinivasan, V. J. Structural and functional human retinal imaging with a fiber-based visible light OCT ophthalmoscope. *Biomed Opt Express* **8**, 323-337, doi:10.1364/BOE.8.000323 (2017).

7 Corwin, K. L. *et al.* Fundamental amplitude noise limitations to supercontinuum spectra generated in a microstructured fiber. *Applied Physics B* **77**, 269-277 (2003).

8 Sorin, W. V. & Baney, D. M. A simple intensity noise reduction technique for optical low-coherence reflectometry. *IEEE Photonics Technology Letters* **4**, 1404-1406 (1992).

9 Mandel, L. Fluctuations of photon beams and their correlations. *Proceedings of the Physical Society* **72**, 1037 (1958).

10 Hodara, H. Statistics of thermal and laser radiation. *Proceedings of the IEEE* **53**, 696-704 (1965).

11 Dudley, J. M., Genty, G. & Coen, S. Supercontinuum generation in photonic crystal fiber. *Reviews of modern physics* **78**, 1135 (2006).

12 Wetzel, B. *et al.* Real-time full bandwidth measurement of spectral noise in supercontinuum generation. *Scientific reports* **2**, 882 (2012).

13 Béjot, P., Kasparian, J., Salmon, E., Ackermann, R. & Wolf, J.-P. Spectral correlation and noise reduction in laser filaments. *Applied Physics B* **87**, 1 (2007).

14 Dorrer, C., Belabas, N., Likforman, J.-P. & Joffre, M. Spectral resolution and sampling issues in Fourier-transform spectral interferometry. *JOSA B* **17**, 1795-1802 (2000).

15 Huang, D. *et al.* Optical coherence tomography. *science* **254**, 1178-1181 (1991).

16 Jensen, M. *et al.* Noise of supercontinuum sources in spectral domain optical coherence tomography. *JOSA B* **36**, A154-A160 (2019).

17 Brown, W. J., Kim, S. & Wax, A. Noise characterization of supercontinuum sources for low-coherence interferometry applications. *J. Opt. Soc. Am. A* **31**, 2703-2710, doi:10.1364/JOSAA.31.002703 (2014).

18 Yao, X., Gan, Y., Marboe, C. C. & Hendon, C. P. Myocardial imaging using ultrahigh-resolution spectral domain optical coherence tomography. *Journal of biomedical optics* **21**, 061006 (2016).

19 Zhang, T., Kho, A. M. & Srinivasan, V. J. Improving visible light OCT of the human retina with rapid spectral shaping and axial tracking. *Biomedical Optics Express* **10**, 2918-2931 (2019).

20 Chong, S. P. *et al.* Ultrahigh resolution retinal imaging by visible light OCT with longitudinal achromatization. *Biomed Opt Express* **9**, 1477-1491, doi:10.1364/BOE.9.001477 (2018).

21 Kho, A. & Srinivasan, V. J. Compensating spatially dependent dispersion in visible light OCT. *Optics letters* **44**, 775-778 (2019).
